# A Growth Mindset Message Leads Parents to Choose More Challenging Learning Activities

**DOI:** 10.3390/jintelligence11100193

**Published:** 2023-10-09

**Authors:** Jing Tian, Grace Bennett-Pierre, Nadia Tavassolie, Nora S. Newcombe, Marsha Weinraub, Annemarie H. Hindman, Kristie J. Newton, Elizabeth A. Gunderson

**Affiliations:** 1 Department of Psychology, Fordham University, Bronx, NY 10458, USA; 2 Department of Psychology and Neuroscience, Temple University, Philadelphia, PA 19122, USA; grace.bennett-pierre@temple.edu (G.B.-P.); nadia.tavassolie@temple.edu (N.T.); newcombe@temple.edu (N.S.N.); marsha.weinraub@temple.edu (M.W.); 3 College of Education and Human Development, Temple University, Philadelphia, PA 19122, USA; annemarie.hindman@temple.edu (A.H.H.); kjnewton@temple.edu (K.J.N.); 4 Department of Psychological and Brain Sciences, Indiana University Bloomington, Bloomington, IN 47405, USA

**Keywords:** growth mindset, home learning environment, spatial skills, literacy, parental beliefs

## Abstract

Prior research has shown that the home learning environment (HLE) is critical in the development of spatial skills and that various parental beliefs influence the HLE. However, a comprehensive analysis of the impact of different parental beliefs on the spatial HLE remains lacking, leaving unanswered questions about which specific parental beliefs are most influential and whether inducing a growth mindset can enhance the spatial HLE. To address these gaps, we conducted an online study with parents of 3- to 5-year-olds. We found that parents’ growth mindset about their children’s ability strongly predicted the spatial HLE after controlling for parents’ motivational beliefs about their children, beliefs about their own ability, children’s age, children’s gender, and family SES. Further, reading an article about growth mindset led parents to choose more challenging spatial learning activities for their children. These findings highlight the critical role of parents’ growth mindset in the spatial HLE. Crucially, these findings demonstrate that general growth mindset messages without specific suggestions for parental practices can influence parental behavior intentions. Further, these effects were also observed in the control domain of literacy, underscoring the broad relevance of the growth mindset in the HLE.

## 1. Introduction

Spatial skills—abilities that involve mentally manipulating objects in space and reasoning about spatial relationships—are strong predictors of STEM achievement and of choices to pursue STEM majors and occupations (Atit et al. 2021; Hsi et al. 1997; Sorby 2009; Tian et al. 2022; Wanzel et al. 2003; Wu and Shah 2004). Early development of spatial skills is consistently predicted by the spatial home learning environment (HLE), including the type and frequency of spatially-relevant home learning activities that parents engage in with their children (Ferrara et al. 2011; Jirout and Newcombe 2015; Levine et al. 2012; Pruden et al. 2011; Pruden and Levine 2017). However, considerable individual and gender differences exist in the spatial HLE (Ho et al. 2018; Levine et al. 2012; Pruden and Levine 2017; Zippert and Rittle-Johnson 2018). Research in other domains, including literacy and math, suggests that variability in the HLE can be partly explained by parents’ beliefs, including motivational beliefs about their child in the specific domain, beliefs about their own abilities in that domain, and growth versus fixed mindsets about intelligence (del Río et al. 2017; Hume et al. 2015; Muenks et al. 2015; Sonnenschein et al. 2012). However, relatively less is known about what types of parental beliefs influence the spatial HLE. Further, causal evidence regarding the relationship between parents’ beliefs and the HLE is limited, leaving open the question of whether the relationships between parents’ beliefs and the HLE are driven by confounding variables. Examining whether changing parents’ beliefs leads to changes in the HLE that benefit children’s development bears both theoretical and practical importance.

In the current study, we aim to address these gaps by asking (1) what parental beliefs (including motivational beliefs about their child, beliefs about parents’ own ability, and growth mindset) explain unique variance in the HLE and (2) whether inducing a growth mindset (via reading an article about how children’s intelligence can be improved through a stimulating environment) leads parents to choose more challenging learning activities for their children. We ask these questions in the spatial as well as the literacy domains, which allows us to assess the extent to which the effects of interests are domain-specific versus domain-general. Additionally, we examine whether the effect of growth mindset induction in each domain depends on gender, as parents hold gender-stereotyped beliefs in both domains but in opposite directions. Parents often report stronger motivational beliefs for boys than girls in the spatial domain and stronger motivational beliefs for girls than boys in the verbal domain (Baroody and Diamond 2013; Levine et al. 2012; Nichols 2002; Pruden et al. 2011; Vasilyeva et al. 2021). Finally, we test a new measure of the spatial HLE that assesses parents’ familiarity with specific spatial toys and activities, which we expect to be less impacted by social desirability bias compared to parents’ self-report of the frequency of home learning activities.

### 1.1. Spatial Skills and the Spatial Home Learning Environment

Spatial skills are essential for learning many STEM subjects, including mathematics, chemistry, and engineering (Atit et al. 2021; Sorby 2009; Wu and Shah 2004). For example, a recent meta-analysis found that spatial skills and math achievement are moderately correlated (*r* = 0.36) throughout development (Atit et al. 2021). Further, students with strong spatial skills are more likely to pursue STEM fields (Shea et al. 2001; Tian et al. 2022; Wai et al. 2009; Webb et al. 2007). Analysis of a national longitudinal dataset reveals that 4th-grade spatial skills directly predicted choosing STEM majors in college, above and beyond math achievement and motivation, verbal achievement and motivation, and family background (Tian et al. 2022). 

In spatial skills, sizable individual differences and gender differences (in mental rotation) emerge in early childhood (Hegarty and Waller 2005; Hyde 2016; Lauer et al. 2019). Individual differences in spatial skills persist over time starting in early childhood, such that early spatial skills predict spatial skills later on (Mix and Cheng 2012; Verdine et al. 2017). While the existence and size of gender differences vary across different types of spatial skills (Atit et al. 2013; Nazareth et al. 2019; Newcombe 2020; Voyer et al. 1995), a recent meta-analysis shows that a significant male advantage in mental rotation emerges by six years of age and continues to widen at least through early adulthood (Lauer et al. 2019). 

One factor that contributes to both individual and gender differences in spatial skill is the spatial HLE, such as engaging in spatial play with blocks and puzzles (Ferrara et al. 2011; Levine et al. 2012; Thomson et al. 2018; Verdine et al. 2014). Spatial play provides ample opportunities for children to learn spatial words (e.g., square, curve, and pointy), practice mental rotation, and work on spatial planning (Ferrara et al. 2011; Levine et al. 2012; Thomson et al. 2018; Verdine et al. 2014). For example, controlling for parent education and income, children who engaged in puzzle play during home observations between two and four years of age showed stronger mental transformation skills when they were 4.5 years old than those who did not play with puzzles (Levine et al. 2012). In a large sample of two- to seven-year-olds, greater parent-reported frequency of children’s spatial activities (blocks, puzzles, and board games) was associated with children’s higher performance on a block design task, a measure of spatial skill, after controlling for other cognitive abilities (Jirout and Newcombe 2015). Moreover, some evidence suggests that boys engage in more spatial play and receive higher quality spatial support from their parents than girls, which might contribute to the early male advantage in spatial skills (Jirout and Newcombe 2015; Levine et al. 2012; Pruden and Levine 2017). 

### 1.2. Literacy and the Literacy Home Learning Environment

Literacy, the ability to read and write, is essential for acquiring new knowledge and functioning in modern society. Around 3 years of age, children start to develop *emergent literacy*, which includes a range of knowledge, skills, and attitudes, such as vocabulary, phonological awareness, oral narrative skills, and print motivation (Teale and Sulzby 1986; Whitehurst and Lonigan 1998). These early knowledge and skills are intercorrelated and exhibit considerable stability throughout development (Dickinson et al. 2003; Lonigan et al. 2000). Critically, emergent literacy skills are precursors to formal reading and writing abilities (Dickinson et al. 2003; Reese et al. 2010; Suggate et al. 2018; Whitehurst and Lonigan 1998). For example, a recent longitudinal study found that children’s vocabulary at 19 months and oral narrative skills around school entry predicted their reading comprehension in adolescence (Suggate et al. 2018). 

Gender differences favoring girls in emergent literacy and literacy skills have been widely documented, both in the U.S. and internationally (Chiu and McBride-Chang 2006; Deasley et al. 2018; Klecker 2006; McTigue et al. 2021; Vasilyeva et al. 2021). Analyses of 4th, 8th, and 12th graders’ reading scores on the U.S. National Assessment of Educational Progress from 1992 to 2003 found that girls outperformed boys in all grade levels and years of testing (Klecker 2006). In the 2000 Program for International Student Assessment (PISA), 15-year-old girls from all 43 participating countries outperformed their boy counterparts on reading achievement (Chiu and McBride-Chang 2006). Some studies have shown that this female advantage in literacy appears as early as 4 years of age (Deasley et al. 2018; McTigue et al. 2021).

The literacy HLE, specifically parent-child shared book reading, plays an important role in children’s early literacy development (Blewitt et al. 2009; Dickinson et al. 2012; Sénéchal et al. 1996; Sénéchal and LeFevre 2002). Shared book reading offers opportunities for children to be exposed to story structures, learn new words, and engage in conversations about and beyond the story text. Storybook exposure at home is associated with young children’s vocabulary, listening comprehension, and phonological awareness (Farrant and Zubrick 2012; Manolitsis et al. 2013; Sénéchal et al. 1998; Sénéchal and LeFevre 2002). A longitudinal study revealed that storybook exposure at home when children were in kindergarten was not only associated with their contemporaneous receptive vocabulary, but also with growth in vocabulary between kindergarten and Grade 1 (Sénéchal and LeFevre 2014). Further, parents of girls reported stronger literacy interest of their children and reported engaging their children in more literacy activities than parents of boys, and these differences might contribute to girls’ early advantage in literacy skills (Baroody and Diamond 2013; Vasilyeva et al. 2021).

### 1.3. Factors Influencing the Spatial and Literacy Home Learning Environment

The pivotal role of the HLE in children’s early cognitive development, along with large variations across families in it, underscores the importance of identifying malleable factors contributing to a high-quality HLE. Eccles and colleagues theorized that parents’ roles in constructing the HLE involve them being both *role models* and *expectancy socializers* (Eccles Parsons et al. 1982). While engaging in home learning activities, parents influence children through behaviors consistent with parents’ own abilities (i.e., as role models) and with parents’ motivational beliefs about their children (i.e., as expectancy socializers). The theory has received support from several studies (Berkule et al. 2008; Deckner et al. 2006; del Río et al. 2017; Elliott et al. 2017; Hume et al. 2015; Zippert and Rittle-Johnson 2018). For example, adolescents’ time spent on reading positively related to their parents’ time spent on reading (Mullan 2010). In another study, parents who had higher expectations for children’s numeracy abilities and lower levels of math anxiety more often engaged their children in numeracy activities (del Río et al. 2017). In the spatial domain, Zippert and Rittle-Johnson (2018) reported parents’ beliefs about their own spatial abilities to be associated with the spatial HLE.

Another important factor that may influence the HLE is parents’ growth mindset. The belief that intelligence is malleable and can be improved through effort is called a *growth mindset*, which contrasts with a *fixed mindset*—the belief that intelligence is pre-determined and not under one’s own control (Dweck 2006). Parents who had stronger growth mindsets reported more frequent engagement in learning activities in hypothetical interactions with their preschool-aged children (Muenks et al. 2015). Several parent interventions that involved promoting growth mindset have led to changes in parental behaviors (Moorman and Pomerantz 2010; Rowe and Leech 2019) or child outcomes (Andersen and Nielsen 2016; Rowe and Leech 2019). In one study, mothers of early elementary school children received a short message on either how a specific test (i.e., Raven’s Progressive Matrices) assesses children’s intellectual potential (i.e., growth mindset induction) or how the test assesses children’s innate intelligence (Moorman and Pomerantz 2010). During the same experimental session, mothers who received the growth mindset message exhibited more autonomy-supportive (rather than controlling) behaviors when working on Raven’s Progressive Matrices problems with their children. In another intervention, parents of 10-month-olds were encouraged to use pointing gestures when playing with their children. Parents also received a growth mindset message about the malleability of children’s language development and how parents can help their children’s language grow by pointing and encouraging their children to point (Rowe and Leech 2019). Parents in the control condition did not receive any of these messages. All parents were then given age-appropriate toys to play with their children over two months. When children were 12 months old, both parents and children in the intervention condition used more pointing gestures than those in the control condition, although the condition did not affect children’s vocabulary. In a large-scale randomized control trial, parents of second graders in the intervention group received booklets and videos with growth mindset messages about the malleability of children’s reading development and were encouraged to read with their children (Andersen and Nielsen 2016). No treatment was given to parents in the control group. At both two and seven months after the intervention, children whose parents received the intervention showed stronger reading and writing achievement than children in the control condition. The effectiveness of these interventions, despite considerable variance in the intervention length and child ages across studies, suggests that parents’ growth mindset may be a crucial factor in children’s cognitive development. However, all three interventions either used a growth mindset message specific to the parent-child interaction activity or coupled it with specific suggestions for parenting practices, making it unclear whether a general growth mindset message alone will yield positive changes in parental behavior.

Despite substantial work examining the associations between multiple aspects of the parents’ beliefs and the HLE, to our knowledge, no study to date has simultaneously examined parents’ beliefs about their own abilities, parents’ motivational beliefs about their child, and parents’ growth mindset. This gap in the literature leaves open the question of which aspect of parents’ beliefs most strongly predicts the HLE independent of other aspects. Further, there has been no research, to our knowledge, on how parents’ growth mindset is associated with the spatial HLE and no causal evidence regarding the relationship between parents’ beliefs and the spatial HLE. Therefore, it is unclear whether the spatial HLE can be improved through interventions on parents’ beliefs. Answering these questions is critical for developing parent interventions to improve the spatial HLE. 

The current study sought to address these gaps and advance prior research in several ways. First, we conducted a comprehensive survey of the parental beliefs that might influence the spatial and literacy HLE. This encompasses parents’ beliefs about their own ability, parents’ motivational beliefs about their children (i.e., child’s ability, interest, importance of child’s ability), and growth mindset about their child’s ability. 

Second, we explored whether inducing parents to have a growth mindset leads to changes in the HLE, especially in counter-gender-stereotyped domains. We focused on one specific aspect of parents’ behavior change—the level of challenge of the home learning activities they would choose for their child. We expected that inducing parents to believe that intelligence is malleable would lead them to value the learning opportunities afforded by home learning activities and choose to engage their children in more stimulating ways (i.e., by choosing harder tasks). Further, we expected this effect to be more pronounced in counter-gender-stereotyped domains, with the growth mindset message having a greater effect in the spatial domain for parents of girls and a greater effect in the literacy domain for parents of boys. This expectation is based on the assumption that parents initially hold gender-stereotyped beliefs and tend to choose less challenging activities for their child in gender-counter-stereotyped domains, as suggested by prior work (Baroody and Diamond 2013; Levine et al. 2012; Nichols 2002; Pruden et al. 2011; Vasilyeva et al. 2021). Increasing parents’ belief that intelligence is malleable may, therefore, be particularly impactful in counter-gender-stereotyped domains, where parents may initially not believe that their child can do well. Evidence supporting this expectation includes research showing that endorsing a growth mindset is associated with higher academic performance, particularly for youth facing stereotypes threats in those fields (Good et al. 2003).

Finally, we developed and tested a new measure of the spatial HLE—the Spatial Toys and Activities Checklist (STAC). This measure is modeled after research in the literacy domain, in which the Children’s Title Checklist (CTC) was developed to reduce potential social desirability bias associated with reporting frequency of home literacy activities (Sénéchal et al. 1996). The CTC, which asks parents to select titles of children’s books that they recognize in a list of real titles and plausible foils, is believed to reflect parents’ familiarity of children’s literature resulting from reading with their children (Sénéchal et al. 1998). In prior research, the CTC better predicted children’s vocabulary, listening comprehension, and phonological awareness than parents’ self-reports of the literacy HLE (Sénéchal et al. 1996, 1998; Sénéchal and LeFevre 2002). To capture the spatial HLE in a similar way, we developed a checklist of popular children’s spatial toys and activities and tested its convergent and divergent validity. With these aims, we asked three research questions (RQs).

RQ1. What parent beliefs contribute to the spatial and the literacy HLE? 

RQ2. Does reading an article promoting a growth mindset about intelligence lead parents to choose more challenging activities for their children?

RQ3. Is the effect tested in RQ2 stronger in a counter-gender-stereotyped domain than a gender-stereotyped domain?

## 2. Methods

### 2.1. Participants

Parents of 3- to 5-year-old children were recruited from Amazon Mechanical Turk (MTurk). A total of 1771 MTurk workers completed a screening survey that included a question asking about the age of their children, if any, along with several filler questions about irrelevant personal information. Several measures were taken to ensure the quality of data. We first excluded those who took the screening survey multiple times (*N* = 49) and those who were potentially bots based on IP address (*N* = 75). Our determination of fraudulent IP address was affirmed by analyses using the *R* package *rIP* (Waggoner et al. 2019). Among the remaining 1647 MTurk workers, 762 reported having at least one child aged 3- to 5-years-old, and 199 of them consented to participate in the full study. 

Among the 199 participants, 22 were excluded due to a technical error in the setup of the survey. We excluded another 58 participants who did not pass quality checks (e.g., reported their own gender differently in the screening survey and demographic questionnaire), attention checks (i.e., answered more than half of the attention check questions incorrectly), or manipulation checks (see details below). More details about participant exclusion are reported in the Supplementary Materials, Section A. 

Our analytic sample consisted of 120 participants. Based on participants’ reports (*N_race_* = 120), 74% were White, 12% were Black or African American, 11% were Asian or Asian American, and 3% were multiracial. Five percent of participants (*N_ethnicity_* = 120) identified as Spanish/Hispanic/Latino ethnicity, while 95% did not. The maximum years of education averaged 15.76 years (where 16 years is equivalent to a Bachelor’s degree; SD = 1.89, *N_education_* = 120). Family income averaged USD 69,479 (*SD* = USD 27,852, *N_income_* = 120). The study was approved by the Temple University Institutional Review Board (IRB) under protocol 24531, “The Development of Academic Skills and Motivation: The Role of the Home Environment.”

### 2.2. Procedure

After providing consent to participate, participants were asked to report the age and gender of their 3- to 5-year-old child and to answer all questions in the study thinking about this child. If participants had multiple children in this age range, they were asked to report information for the oldest child in the age range and to think about that child throughout the study. 

Participants then completed parallel questionnaires in the spatial and literacy domains. These questionnaires asked about the HLE, motivational beliefs about their child (about child’s ability, interest, and importance of child’s ability), beliefs about their own ability, and growth mindset about their child’s ability. Subsequently, participants were randomly assigned to read a *Psychology Today*-style article on the malleability of intelligence (the growth mindset condition) or on déjà vu (the control condition). After reading the article, participants completed manipulation checks and questionnaires on learning activity choices, considerations for activity choice, and growth mindset about their child’s ability. The questionnaires on learning activity choices and growth mindset included both spatial and literacy questions. 

All participants received the measures in the same order: HLE checklist, HLE questionnaire, motivational beliefs about child, beliefs about own ability, growth mindset, reading an article on growth mindset or a control article about déjà vu, learning activity choice, learning activity considerations, and growth mindset. On the learning-activity-choice measure, spatial and literacy questions were combined in the same questionnaire. Participants received this learning-activity-choice questionnaire in one of two pseudo-random orders, with the constraint that no more than two consecutive questions concerned activities in the same domain. On all other measures that had both spatial and literacy questionnaires, half of the participants received literacy questionnaires first, and the other half received spatial questionnaires first. Finally, participants completed a demographic questionnaire. Participants completed the study on Qualtrics. All study questionnaires are presented in the Supplementary Materials (Sections B–E).

### 2.3. Growth Mindset Induction

Participants assigned to the growth mindset condition read a counterfeit article on the malleability of intelligence adapted from prior research (Bergen 1991; Schroder et al. 2014). The article describes “recent research” on how intelligence can be improved via a stimulating environment. Participants assigned to the control condition read a genuine article describing recent research findings on déjà vu (Cleary 2018). Both articles were of similar length, readability level, and format. Participants were asked to read the article carefully and remember the main points in the article for a short memory test right after (i.e., manipulation check).

After reading the article, participants were asked to summarize the main points of the article without the article being present. They were asked to rate how difficult the article was to understand, how credible they found the article to be, how persuasive they found the article to be, and how much they agreed with the views expressed in the article on 7-point Likert scales. On all the Likert scales used in this study, unless noted otherwise, higher values represented stronger agreement with the statement.

Participants’ summaries of the growth mindset induction or the control articles were independently coded by two researchers who were unaware of the participants’ assigned conditions. The researchers coded whether the summary was consistent with ideas in either article, and if so, which article it was. Intercoder reliability was 84%, and all disagreements were resolved through discussion. The first author then examined whether the article that the coders judged each participant had read was consistent with the participant’s assigned article. Our analytic sample only included participants whose summaries were coded as consistent with the article they were assigned to read. Twenty-three participants were excluded based on this criterion. Excluding participants who failed the manipulation checks allowed us to examine the manipulation effect among those who processed the manipulation as intended.

### 2.4. Pre-Induction Measures

#### 2.4.1. HLE

The spatial HLE was measured by a children’s Spatial Toys and Activities Checklist (STAC; Supplementary Materials, Section C) and a spatial HLE questionnaire. We developed the STAC the based on Sénéchal et al.’s (1996) Children’s Title Checklist (CTC). The STAC comprised 40 names of real spatial toys and activities appropriate for young children (such as DUPLOs, Tangrams, and Lincoln Logs) and 20 fictitious names (such as Linderhop, Kangablox, and Snapweez). Participants were asked to check the names of popular spatial toys and activities that they recognized, without guessing since some names were not real. Performance on the STAC was indexed by the proportion of chosen real names minus the proportion of chosen foil names. We calculated split-half reliability of the STAC by dividing it into two lists with the odd-numbered and even-numbered names, respectively. The split-half reliability of the STAC was 0.71.

The spatial HLE questionnaire, adapted from Zippert and Rittle-Johnson (2018), contained six items asking how frequently participants engage in specific spatial activities with their children, such as playing with puzzles and doing mazes. The questions used a 7-point Likert scale, with 1 = never, 2 = once a month, 3 = 2–3 times a month, 4 = 1–2 times a week, 5 = 3–4 times a week, 6 = 5–6 times a week, and 7 = daily. The Cronbach’s alpha for the questionnaire was 0.66, and it increased to 0.71 after excluding the item about experience with computer games or apps. The decision to remove this item was supported by a prior study in which an item on playing computer games loaded negatively on the spatial factor of the HLE (Hart et al. 2016). We calculated composite scores of the questionnaire by averaging responses on each item, excluding the item about computer games and apps.

The literacy HLE was measured using the children’s CTC and a literacy HLE questionnaire, both adapted from Sénéchal et al. (1996, 1998). The CTC included 60 children’s book titles, of which 40 were popular children’s books (such as *A Difficult Day*, *Curious George*, and *Goodnight Moon*) and 20 were foils (such as *Big Old Trucks*, *Terry Toad*, and *Winter Fun on Snowy Days*). Participants were asked to check the titles that they recognized. Participants were told that some titles were made up and that they should not guess. Performance on the checklist was indexed by the proportion of chosen real book titles minus the proportion of chosen foil book titles. We calculated split-half reliability of this checklist using the same method as the STAC, and the split-half reliability of the CTC was 0.70.

The literacy HLE questionnaire asked participants to report the frequency of reading to their children at bedtime and other times, frequency of children’s initiated book reading, frequency of library visits, number of children’s books owned, and the age at which they started reading to their children. For example, one question asked, “At bedtime, how often do you, or other members of the family, read to your child in a typical week?” Because data collection happened during the COVID-19 pandemic, we modified the library-visit question to ask about their experience before the pandemic. The frequency questions used the same 7-point Likert scale as the spatial HLE questionnaire. The response options for the number of children’s books owned were on a 6-point scale, with 0 = none, 1 = 1–20, 2 = 21–40, 3 = 41–60, 4 = 61–80, and 5 = more than 80. We reverse coded the age that children started reading so that larger values indicated a longer duration of literacy experience. We then converted responses to each item to *z*-scores so that scores of different items on this questionnaire were on similar scales. The internal consistency of the full questionnaire was acceptable (Cronbach’s alpha = 0.71), and it increased to 0.78 after excluding the question about library visits. Because of this, and because unlike other questions, the library visit question asked about past instead of current home literacy experience, we excluded the library visit question when calculating composite scores from the questionnaire. Composite scores were calculated by averaging the *z*-scores of each item.

#### 2.4.2. Beliefs about Child’s Ability, Interest, and Importance of Child’s Ability

We measured participants’ motivational beliefs about their child using questions adapted from Zippert and Rittle-Johnson (2018). Beliefs about child’s ability, child’s interest, and importance of child ability in the spatial and literacy domains were each measured with one question using 7-point Likert scales. For example, beliefs about child’s spatial ability were measured using the question, “How good is your child in spatial activities?” 1 = not good at all, and 7 = very good. To contextualize the questions, participants were presented with a few examples of activities in the spatial and literacy domains. Specifically, examples were “doing puzzles and building with blocks” for spatial activities and “reading story books and telling stories” for literacy activities.

#### 2.4.3. Belief about Own Ability

Participants’ beliefs about their own spatial ability were measured using the Philadelphia Spatial Abilities Scale (Hegarty et al. 2010), which consisted of 16 statements about abilities, preferences, and experiences in completing various small-scale spatial tasks. Examples of statements include “I can easily visualize my room with a different furniture arrangement” and “I am good at putting together furniture with only the use of diagrams.” Participants indicated how much they agreed with each statement on 7-point Likert scales. We reverse coded two negatively framed items so that a higher score corresponded to stronger beliefs in one’s spatial abilities. The internal consistency for this scale was excellent (α = 0.91). Composite scores were calculated by averaging responses to each item. 

Participants’ beliefs about their own literacy ability were measured using the Philadelphia Verbal Abilities Scale (Hegarty et al. 2010), which included 10 statements about literacy abilities, preferences, and experiences. Examples of the statements include “I am good at crossword puzzles” and “I can easily follow a complex verbal argument.” Participants indicated their level of agreement with each statement using a 7-point Likert scale. Three negatively framed items on the scale were reverse coded so that a higher score indicates stronger beliefs in verbal abilities. The internal consistency for this scale was good (α = 0.78). Composite scores were calculated by averaging responses to each item.

#### 2.4.4. Growth Mindset about Child

Participants’ growth mindset about their child was measured using a scale adapted from Muenks et al. (2015), which included six statements about the fixedness of their child’s spatial abilities and six statements about the fixedness of their child’s literacy abilities. For example, one statement about spatial abilities said, “My child’s spatial ability is innate and will never change”, and another said “My child’s spatial ability can only be substantially improved during a specific period of time in his/her development.” Participants indicated how much they agreed with each statement on a 7-point Likert scale. Four negatively framed items in each domain were reverse coded so that higher scores reflected stronger growth mindsets. The internal consistency of the measure was good (spatial, α = 0.82; literacy, α = 0.84). Composite scores were calculated by averaging responses across items within each domain.

### 2.5. Post-Induction Measures

#### 2.5.1. Learning Activity Choice

**Figure 1:**
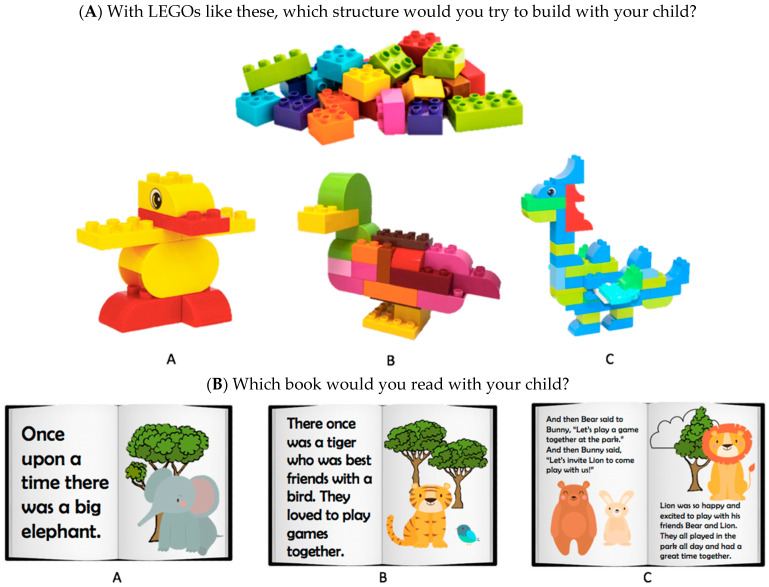
Example questions about learning activity choices in the (**A**) spatial and (**B**) literacy domains. In both examples, option (**a**) is easy, (**b**) is medium, and (**c**) is hard. During the study, the order of easy, medium, and hard options was randomized for each item.

The measure of learning activity choice was designed to measure the level of challenge that participants intended for their children when engaging in spatial and literacy learning activities, and it served as our primary outcome of interest. Participants were presented with six spatial and six literacy activities. For each activity, they were asked to choose what they would do with their child from three options that varied in difficulty (for all items, see Supplementary Materials, Section E). The criterion we used to determine difficulty depends on the activity. For example, one spatial question showed a pile of LEGO blocks and asked, “With LEGOs like these, which structure would you try to build with your child?” The three choices were LEGO structures that varied in the number of blocks needed, ranging from easy to hard (Figure 1A). One literacy question asked, “Which book would you read with your child?” The three choices were illustrations of the inside of made-up children’s story books that varied in the number of words and number of characters involved in the story (Figure 1B). See Supplementary Materials, Section F for the criteria we used to determine difficulty for each activity. The order of the three choices was randomized for each question. We coded choices with numeric values, with 1 = easy, 2 = medium, and 3 = hard. Internal consistency of the scale was good for spatial items (α = 0.77) and moderate for literacy items (α = 0.65). Composite scores were separately calculated for spatial and literacy items by averaging responses to items in the corresponding domain.

#### 2.5.2. Growth Mindset about Child

Participants completed the same growth mindset measures as in the pre-induction phase, separately in the spatial and literacy domains. The internal consistency of the measure post-induction was good (spatial, α = 0.84; and literacy, α = 0.87).

#### 2.5.3. Activity Choice Considerations

To better understand the potential effects of growth mindset induction beyond the level of challenge of learning activity choices, we included a measure that examined various aspects of parents’ considerations when choosing children’s learning activities. Participants rated the importance of different factors when choosing activities for their child, including the child’s enjoyment, their own enjoyment, the level of difficulty for the child, the level of difficulty for themselves, and how much the child would learn. Participants then completed a question about domain-general activity difficulty, where they indicated the level of challenge they would choose when playing toys and games with their child on a 0 to 100 sliding scale, with 0 being “very easy” and 100 being “very hard”. This question was not specific to the spatial or literacy domains. Lastly, participants rated the importance of playing at home, parents’ teaching, and learning from school for improving their child’s literacy and spatial ability. Ratings of importance were on a 7-point Likert scale, with 1 being “not at all important” and 7 being “extremely important”.

### 2.6. Missing Data

On each measure, composite scores were calculated with available data where a participant had answered at least half of the items on that measure. If a participant omitted more than half of the items on a measure, their composite score on that measure was recorded as missing. Fewer than 2.5% of responses were missing on any item, and 0.4% of all composite scores were missing. For each analysis, we included all the participants who had data available on the measures used in that analysis.

## 3. Results

### 3.1. Preliminary Analyses

#### 3.1.1. Manipulation Checks

Participants in both conditions found that the articles were not too difficult to understand (1 = not difficult to understand at all and 7 = very much difficult to understand; *M_growth_* = 2.23, *SD_growth_* = 1.64; *M_control_* = 3.17, *SD_control_* = 1.58), very credible (1 = not credible at all and 7 = very much credible; *M_growth_* = 5.85, *SD_growth_* = 1.05; *M_control_* = 5.42, *SD_control_* = 1.26), very persuasive (1 = not persuasive at all and 7 = very much persuasive; *M_growth_* = 5.72, *SD_growth_* = 1.05; *M_control_* = 5.20, *SD_control_* = 1.31), and they agreed a lot with views expressed in the articles (1 = not agree at all and 7 = agree very much; *M_growth_* = 5.62, *SD_growth_* = 1.11; *M_control_* = 5.27, *SD_control_* = 1.11). Participants in the growth mindset induction condition found the article less difficult to understand, more credible, and more persuasive than participants in the control condition (*p*s < .05). Participants from the two conditions did not significantly differ in their agreement with the views expressed in the articles (*p* = .086). Excluding participants who failed the manipulation checks allowed us to examine the manipulation effect among those who processed the manipulation as intended.

#### 3.1.2. Condition Differences

Table 1 shows descriptive statistics of demographic characteristics and all measures by condition (see Supplemental Materials, Section G, for histograms of participants’ responses on the STAC, the CTC, the HLE questionnaires in the spatial and literacy domains, and the learning activity choice measures in the spatial and literacy domains). T-tests showed that participants in the two conditions did not significantly differ on any of the pre-induction measures. Post-induction, participants in the growth mindset condition chose more challenging spatial activities than those in the control condition, *t* (116.28) = −2.52, *p* = .013, Cohen’s *d* = 0.46. Compared to participants in the control condition, participants in the growth mindset condition thought it was less important to consider how difficult a task was for parents when choosing activities for children, *t* (116.85) = 2.39, *p* = .018, Cohen’s *d* = −0.44. None of the demographic characteristics or any other measures differed by condition.

#### 3.1.3. Correlations

**Figure 2:**
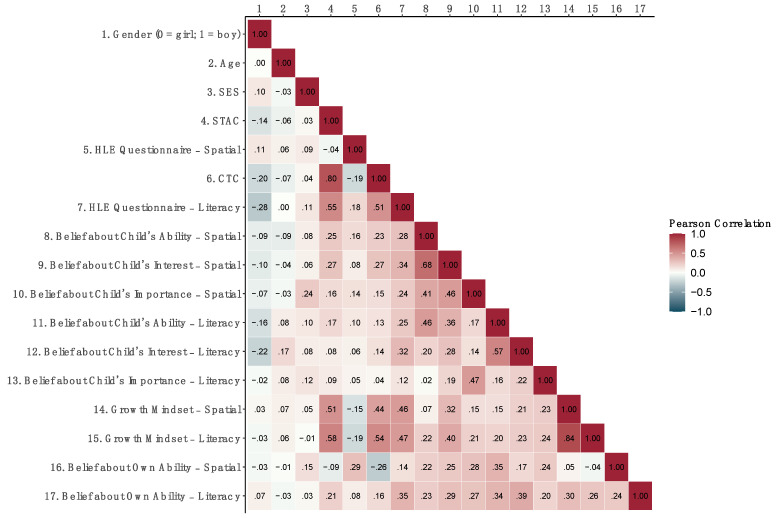
Correlations (Pearson’s *r*) among demographics and all pre-induction measures (with pairwise complete observations). SES stands for socioeconomic status and was a composite variable created by confirmatory factor analysis with maximum parental education and family income. HLE stands for home learning environment. STAC stands for Spatial Toys and Activities Checklist. CTC stands for Children’s Title Checklist. Correlations with Pearson’s |*r|* ≥ .19 are significant.

Figure 2 shows correlations among all demographic characteristics and pre-induction measures. The only pre-induction measures that differed by gender were the CTC, the literacy HLE questionnaire, and participants’ beliefs about children’s literacy interest. Parents of girls provided richer literacy HLE and believed that their children were more interested in literacy activities than parents of boys. 

The two literacy HLE measures (i.e., the CTC and the questionnaire) significantly correlated with each other (*r* (115) = .51, *p* < .001), as one would expect. However, the two spatial HLE measures, the STAC and the spatial HLE questionnaire, did not (*r* (118) = −.04, *p* = .657), suggesting that these two measures were not assessing the same aspects of the spatial HLE. One reason might be that we focused on puzzles and construction toys when designing the STAC, because most prior research on the spatial HLE has focused on these types of activities and because these activities predict children’s spatial development (Ferrara et al. 2011; Jirout and Newcombe 2015; Levine et al. 2012). In contrast, the spatial HLE questionnaire consisted of items besides puzzles and construction toys, including maps, mazes, and connect-the-dot activities. Consistent with this possibility, STAC scores positively correlated with the items measuring participants’ reported frequency of playing with puzzles, *r* (118) = .23, *p* = .012; and construction toys, *r* (118) = .24, *p* = .007, on the questionnaire. However, STAC scores did not correlate with or negatively correlated with reported frequency of doing mazes, *r* (118) = −.08, *p* = .387; doing connect-the-dot activities, *r* (118) = −.18, *p* = .044; or drawing maps or plans, *r* (117) = −.36, *p* < .001. See Supplementary Materials, Section H, for correlations among all items on the spatial HLE questionnaire and STAC.

**Figure 3:**
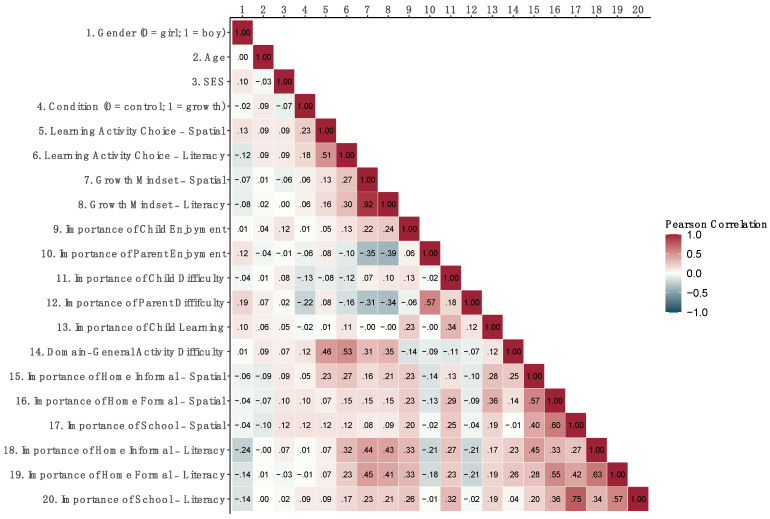
Correlations (Pearson’s *r*) among demographics, condition, and all post-induction measures (with pairwise complete observations). SES stands for socioeconomic status and was a composite variable created by confirmatory factor analysis with maximum parental education and family income. Correlations with Pearson’s |*r|* ≥ .19 are significant.

Figure 3 shows correlations among all post-induction measures. As expected, responses to the domain-general activity difficulty question, which explicitly asked participants to rate the level of difficulty that they would choose for their children’s activities on a sliding scale, moderately correlated with the challenge level of the learning activity choice measure (where participants chose pictures showing ways of engaging in specific learning activities) in both the spatial (*r* (117) = .46, *p* < .001) and the literacy (*r* (117) = 0.53, *p* < .001) domains. There was also a small but significant correlation between the challenge level of learning activity choices and participants’ ratings of the importance of informal home activities for improving children’s skills (spatial, *r* (116) = .23, *p* = .012; and literacy, *r* (116) = .32, *p* = .003).

### 3.2. Main Analyses

RQ1. What parent belief factors contribute to the spatial and the literacy HLE?

To examine factors contributing to the HLE, we conducted multiple linear regressions separately for each of the literacy and spatial HLE measures (Table 2). Predictors in each regression included demographic variables (i.e., child gender, child age, and family SES), participants’ motivational beliefs about their child (i.e., about child ability, child interest, importance of child ability), growth mindset about their child, and beliefs about their own ability. Models predicting the spatial HLE included predictors specific to the spatial domain, and models predicting the literacy HLE included predictors specific to the literacy domain. Table 2 shows results of these regression models. For both literacy HLE measures, girls had a richer literacy HLE and parents’ stronger growth mindset about their child’s literacy ability was associated with a richer literacy HLE. Participants who had stronger beliefs about their own literacy ability also reported more frequent literacy home learning activities with their children on the HLE questionnaire.

Factors contributing to the spatial HLE were less clear and differed across the STAC and the HLE questionnaire. Parents who had stronger beliefs about their own spatial ability reported higher frequency of spatial play on the HLE questionnaire but scored lower on the STAC. Further, parents’ growth mindset about their child’s spatial ability and beliefs about their child’s spatial ability positively predicted scores on the STAC but not on the spatial HLE questionnaire.

RQ2. Does reading a growth mindset message lead parents to choose more challenging activities for their children?
RQ3. Is the effect tested in RQ2 stronger in a gender counter-stereotyped domain than a gender-stereotyped domain?

We tested RQ2 and RQ3 together by examining whether reading the growth mindset article led participants to choose more challenging activities, especially in gender counter-stereotyped domains. To do so, we fit stepwise mixed-effect regression models predicting the level of challenge of learning activity choice, with participants as random effects^1^. We entered condition (growth mindset vs. control), domain (spatial vs. literacy), and demographic factors (child gender, SES, and child age) first. Condition was a between-subjects factor, and domain was a within-subjects factor. In these models, a significant effect of condition, if any, indicates that the activity choice differed between participants in the growth mindset and the control condition; and a significant effect of domain, if any, indicates that the level of challenge of activity choice differed in the spatial and literacy domain. 

In preliminary analyses, the model that included interactions among condition, domain, and child gender did not significantly differ from the model without these interactions, $\chi^{2}\left(4\right)=8.36,p=.079.$ This suggests that the effect of condition on activity choice did not differ across the spatial and literacy domains, nor by the combination of domain and child gender, failing to support the hypothesized effect in RQ3. We, therefore, only included main effects of each of these factors in all subsequent models. As a robustness check, we also ran the models reported below (Models 1–4) separately for the spatial and literacy domains. These models all yielded a main effect of the manipulation condition, with participants from the growth mindset condition choosing more challenging activities than participants from the control condition (see Supplementary Materials, Section I for details of these models).

In the first step (Table 3, Model 1), condition significantly predicted activity choice, β=0.41, p=.009, with participants in the growth mindset condition choosing more challenging activities for their children than participants in the control condition across the spatial and literacy domains, consistent with the hypothesized effect in RQ2. 

In the second step, we asked whether the effect of condition on activity choice would remain when controlling for pre-induction HLE measures, including the questionnaires and checklists. The model that included interactions between the questionnaire and domain and between the checklist and domain did not significantly differ from the model without these interactions, $\chi^{2}\left(2\right)=1.30,p=.521.$ We thus excluded these interactions in this (Table 3, Model 2) and the subsequent models. Condition remained a significant predictor of activity choice. The effects of the pre-induction HLE were also significant, where participants who reported richer HLE on the questionnaires and who scored higher on the checklists in a given domain chose more challenging activities for their children in that domain (HLE questionnaire, β=0.17, p=.004; checklist, β=0.16, p=.030). 

In the third step, we further added participants’ pre-induction motivational beliefs about their child and growth mindset for their child as predictors. The model that included interactions between each of these newly added variables and domain did not significantly differ from the model without these interactions, $\chi^{2}\left(4\right)=8.16,p=.086.$ We thus excluded these interactions in this (Table 3, Model 3) and the subsequent model. Condition remained a significant predictor, and none of these belief measures significantly predicted activity choice. The HLE questionnaire remained a significant predictor (β=0.16, p=.009), but the checklist no longer predicted activity choice (β=0.12, p=.140). 

In the last step, we added participants’ beliefs about their own ability as a predictor. The model with an interaction between participants’ beliefs about their own ability and domain differed from the model without this interaction, $\chi^{2}\left(1\right)=7.29,p=.007.$ We thus included this interaction term (Table 3, Model 4). Condition continued to have a significant effect on activity choice, β=0.38, p=.011, suggesting that participants receiving the growth mindset message chose more challenging activities than participants in the control condition across the two domains.

To understand the interaction between domain and participants’ beliefs about their own ability in Model 4, we fit linear regression models predicting activity choice separately for the spatial (Table 4, Model 5) and literacy (Table 4, Model 6) domains. The predictors included condition, child gender, SES, child age, HLE questionnaire, HLE checklist, pre-induction growth mindset, motivational beliefs about child, and beliefs about own ability. In both the spatial and literacy domains, condition significantly predicted activity choice (spatial: β=0.47, p=.011; literacy: β=0.33, p=.040.) Participants’ beliefs about their own ability were a significant positive predictor of activity choice in the literacy domain, β=0.42, p<.001; but not the spatial domain, β=0.18, p=.076. Additionally, beliefs about the importance of children’s ability significantly positively predicted activity choice in the literacy domain, β=0.19, p=.023; but not the spatial domain, β=−.06, p=.592. None of the other predictors in either model were significant.

## 4. Discussion

The current study aimed to identify specific parental beliefs that uniquely relate to the HLE, with a focus on the spatial HLE. Additionally, we aimed to determine whether the spatial HLE can be improved by reading an article about growth mindset. We also introduced a novel measure of the spatial HLE, the Spatial Toys and Activities Checklist (STAC). Using an online sample of parents of 3- to 5-year-olds, we found that parents’ growth mindset about their child’s ability explained unique variance in the spatial HLE as measured by the STAC, after controlling for parents’ motivational beliefs about their children, beliefs about their own ability, children’s age, children’s gender, and family SES. Further, reading an article about growth mindset led parents to choose more challenging spatial learning activities, even though we found no evidence that reading such an article yielded stronger growth mindsets among parents. We found these effects to also be present in the control (i.e., literacy) domain. However, parents’ responses on the STAC did not correlate with their self-reported frequency of playing spatial toys and games with their children on the spatial HLE questionnaire.

The present findings suggest that parents’ growth mindsets are important contributors to the HLE in both the spatial and literacy domains. Addressing limitations of prior studies, which often examined only a few aspects of parental beliefs (Hume et al. 2015; Muenks et al. 2015; Zippert and Rittle-Johnson 2018), the current study included a relatively comprehensive set of parent beliefs along with growth mindset. Results showed that growth mindset was the parental belief factor that was most robustly related to the HLE. Further, this study, to our knowledge, is the first to show the unique relation of parents’ growth mindset to the spatial HLE. 

The impact of growth mindset beliefs on the HLE was further demonstrated by parents choosing more challenging learning activities after reading about the malleability of children’s intelligence. The effect of the induction remained significant on the level of challenge of learning activity choices even after controlling for a range of parental beliefs, including pre-induction growth mindset. This study extends existing parent interventions on growth mindset to a new aspect of the HLE (i.e., parent choice of learning activities), a new domain (i.e., spatial), and a new age group (i.e., 3- to 5-year-olds; Andersen and Nielsen 2016; Moorman and Pomerantz 2010; Rowe and Leech 2019). Crucially, our findings demonstrate that general growth mindset messages, without explicit suggestions for parental practices, can induce changes in parental behavior intentions. However, an unexpected finding was that the growth mindset induction did not lead to stronger growth mindset beliefs. One possible explanation is that parents already held close-to-ceiling growth mindset beliefs before the induction, and the changes in their beliefs were not captured by the same measure post-induction. Another possibility is that the growth mindset measure was not sensitive to small changes. Nevertheless, the growth mindset induction produced positive changes in parental behavior intentions even among parents with somewhat strong growth mindset beliefs.

We did not find any evidence that the growth mindset induction would have a stronger effect in counter-gender-stereotyped domains. We had predicted such effects based on prior research suggesting parents may hold gendered views of spatial and literacy domains, and that increasing parents’ growth mindset may be particularly impactful in the domains in which parents initially do not believe their child could do well (Baroody and Diamond 2013; Levine et al. 2012; Nichols 2002; Pruden et al. 2011; Vasilyeva et al. 2021). However, it is possible that our participants did not hold strong gender-stereotyped views before the growth mindset induction. Specifically, before the growth mindset induction, the only gender differences were that parents of girls reported stronger beliefs in their child’s literacy interest and a richer literacy HLE than parents of boys. No gender difference emerged in parents’ beliefs about children’s literacy ability, the importance of children’s literacy ability, literacy growth mindset, or any aspects of spatial beliefs and spatial HLE. Another possibility is that parents’ growth mindset about their child might not be domain-specific, as parents’ growth mindsets in the spatial and the literacy domains pre-induction were strongly correlated, even though other parental beliefs between the two domains did not correlate as strongly. Prior research suggests that people hold both global mindset beliefs across multiple ability domains (e.g., math, reading, science) as well as domain-specific mindset beliefs (Gunderson et al. 2017; Lewis et al. 2021; Muenks et al. 2015; Petscher et al. 2017). Further, domain-specific mindset measures, like the ones used in the current study, typically reflect a mixture of both global and domain-specific mindsets (Lewis et al. 2021). Therefore, reading the growth mindset article may have similar effects on parents’ beliefs across domains.

The current findings also contribute to our knowledge of the measurement the spatial HLE. To mitigate the potential social-desirability bias in self-reported frequencies of home learning activities, we developed the Spatial Toys and Activity Checklist (STAC), modeled after the Children’s Title Checklist (CTC; Sénéchal et al. 1996). The STAC exhibited satisfactory reliability comparable to the CTC and strongly correlated with the CTC. However, the STAC did not correlate with the spatial HLE questionnaire, in which parents reported the frequency of engaging in a range of spatial learning activities with their children. Further, different aspects of parental beliefs explained variance in the STAC but not in the spatial HLE questionnaire. These results could mean that parents who are familiar with children’s spatial toys and activities do not necessarily engage their children in these toys and activities more frequently. The results could also suggest that the STAC may capture different aspects of the spatial HLE than the questionnaire. 

We believe the STAC measure may be more effective at capturing aspects of the spatial HLE that benefit children’s spatial development. One reason is that, compared to the spatial HLE questionnaire, the STAC was more closely correlated with the literacy HLE measures and that parents’ growth mindset about their children explained variance in the STAC but not the spatial HLE questionnaire. Another reason is that most prior research on the benefits of early spatial play for children’s development has focused on construction-related activities (Ferrara et al. 2011; Jirout and Newcombe 2015; Levine et al. 2012), which we intentionally emphasized when developing the STAC. This intention was corroborated by the positive correlations between parents’ responses on the STAC and their reported frequencies of playing with puzzles and construction toys but not their reported frequencies of doing maps and plans, mazes, or connect-the-dots activities. Additionally, one prior study found no relationship between first-grade girls’ spatial skills and their mothers’ reports of engagement in a wide range of spatial activities, including many of the items used in the spatial HLE questionnaire used in the current study (Dearing et al. 2012). Future research incorporating direct measures of children’s spatial skills, along with the STAC and spatial HLE questionnaire, is needed. Such research will help clarify the relationship between different measures of the spatial HLE and children’s spatial development as well as the relationship between specific aspects of the spatial HLE and children’s spatial development.

## 5. Limitations

There are several limitations of the current study that should be addressed in future research. First, the study relied on parent-reported intentions of learning activity choice rather than direct behavioral measures, which may not accurately reflect parents’ real-world choices. To address this limitation, future research could record parents’ actual learning activity choices for their children at home after reading about growth mindset. Additionally, the study did not include measures of children’s skills, which hinders our ability to identify which HLE measure better predicts child outcomes. Second, the study used an MTurk sample, which often raises concerns about the quality of responses (Dennis et al. 2020; Waggoner et al. 2019). We are hopeful that our analytic sample did not suffer from this concern as our quality and manipulation checks helped exclude a large number of unusable participants due to fraudulent responses. Further, some research has successfully replicated results from MTurk participants with participants recruited from a university database (Muenks et al. 2015). Nevertheless, future research with parents recruited from off-line venues will help us understand the generalizability of the present findings. Third, even though we tried to match the length and readability of the growth mindset induction and control articles, participants found the growth mindset article easier to read, more credible, and more persuasive. Future research with more closely matched manipulation and control materials will be helpful to distinguish effects led by the messages delivered by the materials from their readability. Finally, the purpose of presenting a growth mindset article in our study was to temporarily induce changes in parental beliefs. While the manipulation successfully resulted in positive changes in parental behavior intentions, achieving meaningful and enduring changes in parental beliefs and behaviors may require the development of interventions through iterative processes. The current findings serve as motivation and offer a promising starting point for the future development of such interventions.

## 6. Conclusions

Despite these limitations, the present findings add to our understanding of HLE of preschoolers in both the spatial and literacy domains. Among a relatively comprehensive set of parental belief measures, parents’ growth mindsets were most strongly associated with both the spatial and literacy HLE. Further, reading about a growth mindset led parents to choose more challenging home learning activities, even among parents with relatively strong growth mindset beliefs initially. These findings hold great promise for interventions aimed at improving children’s spatial skills as they suggest that parents are receptive to growth mindset messages and that they can readily apply the growth mindset beliefs to their own informal home learning activities. 

## Figures and Tables

**Table 1:** Mean and standard deviation of each measure by condition.

Measure	Growth Condition	Control Condition	Growth vs. Control Condition Difference Cohen’s *d*
***Demographics***			
Child Gender	24 parents of girls; 35 parents of boys	26 parents of girls; 35 parents of boys	
Child Age (mo.)	56.11 (8.40)	54.44 (10.24)	0.18
SES	−0.03 (1.06)	0.12 (1.10)	−0.13
***Pre-Induction Measures***			
STAC	0.2 (0.11)	0.2 (0.15)	−0.03
HLE Questionnaire—Spatial	3.19 (1.00)	3.07 (0.97)	0.12
CTC	0.18 (0.14)	0.15 (0.15)	0.21
HLE Questionnaire—Literacy	−0.01 (0.71)	0.01 (0.75)	−0.04
Belief about Child’s Ability—Spatial	5.52 (1.21)	5.51 (1.17)	0.01
Belief about Child’s Interest—Spatial	5.67 (1.31)	5.75 (1.27)	−0.06
Belief about Child’s Importance—Spatial	5.72 (1.28)	5.53 (1.43)	0.14
Belief about Child’s Ability—Literacy	5.57 (1.41)	5.58 (1.33)	0.00
Belief about Child’s Interest—Literacy	5.59 (1.28)	5.59 (1.29)	0.00
Belief about Child’s Importance—Literacy	6.20 (1.12)	6.24 (1.01)	−0.04
Belief about Own Ability—Spatial	4.77 (1.10)	4.71 (1.10)	0.05
Belief about Own Ability—Literacy	5.18 (0.86)	5.07 (0.85)	0.13
Growth Mindset—Spatial	4.62 (1.00)	4.76 (1.03)	−0.14
Growth Mindset—Literacy	4.80 (1.07)	4.77 (1.11)	0.03
***Post-Induction Measures***			
Learning Activity Choice—Spatial	2.11 (0.51)	1.86 (0.53)	0.46 *
Learning Activity Choice—Literacy	2.19 (0.42)	2.03 (0.46)	0.36
Growth Mindset—Spatial	4.98 (0.93)	4.87 (1.01)	0.12
Growth Mindset—Literacy	5.03 (1.03)	4.91 (1.14)	0.11
Important to Choose Activities Based on:			
Child Enjoyment	6.50 (0.79)	6.49 (0.82)	0.01
Parent Enjoyment	3.80 (1.90)	4.02 (1.67)	−0.12
Child Difficulty	5.36 (1.41)	5.69 (1.10)	−0.26
Parent Difficulty	2.62 (1.82)	3.42 (1.86)	−0.44 *
Child Learning	6.10 (0.98)	6.14 (0.98)	−0.04
Domain-General Activity Difficulty (Sliding Scale)	56.93 (18.83)	52.58 (17.77)	0.24
Importance for Improving Child’s Ability:			
Home Informal—Spatial	6.02 (1.13)	5.90 (1.31)	0.10
Home Formal—Spatial	5.74 (1.08)	5.51 (1.30)	0.19
School—Spatial	6.03 (1.15)	5.73 (1.32)	0.24
Home Informal—Literacy	6.47 (0.95)	6.44 (0.90)	0.03
Home Formal—Literacy	6.17 (1.15)	6.19 (0.96)	−0.02
School—Literacy	6.33 (1.14)	6.12 (1.16)	0.18

Note: SES stands for socioeconomic status, and was a composite variable created by confirmatory factor analysis with maximum parental education and family income. HLE stands for home learning environment, STAC stands for spatial toys and activities checklist. Positive Cohen’s *d* values indicate greater values in the growth condition than in the control condition. * *p* < .05.

**Table 2:** Results of regressions predicting the spatial and literacy home learning environment (standardized beta coefficients).

	Spatial HLE		Literacy HLE	
	STAC	Spatial HLE Questionnaire	CTC	Literacy HLE Questionnaire
Intercept	0.19	−0.21	0.24	0.34 **
Child Gender (0 = girl; 1 = boy)	−0.31	0.32	−0.42 *	−0.58 ***
Child Age	−0.07	0.08	−0.09	−0.02
SES	0.03	−0.01	0.07	0.14
Pre-Induction Growth Mindset	0.53 ***	−0.17	0.56 ***	0.40 ***
Belief about Child Ability	0.25 *	0.14	−0.01	−0.002
Belief about Child Interest	−0.07	−0.05	−0.02	0.08
Belief about Child Importance	0.03	0.08	−0.10	−0.06
Belief about Own Ability	−0.17 *	0.26 **	0.05	0.24 **
R-squared	0.36	0.16	0.35	0.38
Adjusted R-squared	0.32	0.10	0.31	0.34

Note: SES stands for socioeconomic status. HLE stands for home learning environment, and STAC stands for spatial toys and activities checklist. Models predicting the literacy HLE included predictors specific to the literacy domain, and models predicting the spatial HLE included predictors specific to the spatial domain. * *p* < .05; ** *p* < .01; *** *p* < .001. VIFs < 2.35.

**Table 3:** Results of mixed-effects regressions predicting challenge level of activity choices (std. beta coefficients).

	Spatial and Literacy Domains (*N* = 120)			
	Model 1	Model 2	Model 3	Model 4
Intercept	−0.22	−0.27	−0.27	−0.25
Condition: growth	0.41 **	0.40 **	0.40 **	0.38 *
Domain: literacy	<0.001	0.01	0.01	0.01
Child gender: boy	0.01	0.11	0.11	0.07
SES	0.12	0.09	0.07	0.07
Child age	0.08	0.08	0.08	0.10
HLE Questionnaire		0.17 **	0.16 **	0.10
Checklist		0.16 *	0.12	0.14
Pre-Induction Growth Mindset			0.06	0.04
Belief about Child Ability			0.14	0.12
Belief about Child Interest			−0.07	−0.10
Belief about Child Importance			0.05	0.06
Belief about Own Ability				0.04
Belief about Own Ability * Domain: literacy				0.27 **
Marginal R-squared	0.06	0.12	0.15	0.19
Conditional R-squared	0.52	0.55	0.55	0.55

Note: SES stands for socioeconomic status. HLE stands for home learning environment. Random effects of participant are included in each model. Marginal R-squared indicates the variances explained by the fixed effects, and conditional R-squared indicates the variances explained by both the fixed and random effects. * *p* < .05; ** *p* < .01.

**Table 4:** Results of linear regressions predicting challenge level of activity choices (std. beta coefficients).

	Spatial Domain	Literacy Domain
	Model 5	Model 6
Intercept	−0.41 *	−0.01
Condition: growth	0.47 *	0.33 *
Child gender: boy	0.30	−0.24
SES	0.07	0.10
Child age	0.08	0.12
HLE Questionnaire	0.08	0.05
Checklist	0.21	0.04
Pre-Induction Growth Mindset	0.08	0.03
Belief about Child Ability	0.07	0.11
Belief about Child Interest	−0.09	−0.13
Belief about Child Importance	−0.06	0.19 *
Belief about Own Ability	0.18	0.42 ***
R-squared	0.18	0.36
Adjusted R-squared	0.09	0.29

Note: SES stands for socioeconomic status. HLE stands for home learning environment. Random effects of participant are included in each model. R-squared indicates the variances explained by the predictors, and adjusted R-squared takes into consideration the number of independent predictors included for explaining the variances. * *p* < .05; *** *p* < .001.

## Data Availability

Data will be available upon request.

## Note

1 We found no evidence that participants in the growth mindset condition held a stronger growth mindset than participants in the control condition after the induction. See Supplementary Materials, Section J, for results of the relevant analyses.

## Supplementary Materials

Supporting information can be downloaded at https://www.mdpi.com/article/10.3390/jintelligence11100193/s1.
