# Supplementary material for: A Growth Mindset Message Leads Parents to Choose More Challenging Learning Activities
Source: J Intell. 2023 Oct 9;11(10):193. doi: 10.3390/jintelligence11100193 (PMC10607904; doi:10.3390/jintelligence11100193)
Supplement: Supplementary file 1 [file jintelligence-11-00193-s001.zip › jintelligence-2362205-supplementary.pdf]

Supplementary Materials

Section A. Participant Exclusion
Section B. Screener Survey
Section C. Pre-Induction Questionnaires
Section D. Manipulation Articles and Manipulation Check
Section E. Post-Induction Questionnaires
Section F. Criteria to Determine Difficulty for Each Activity on the Learning Activity Choice Questionnaire.
Section G. Histograms of participants' responses on the HLE and the Learning Activity Choice measures, By Condition
Section H. Correlations among Items on Spatial Home Learning Environment Questionnaire and Spatial Toys and Activities Checklist
Section I. Results of Regression Models Predicting Learning Activity Choices Separately for the Spatial and Literacy Domains
Section J. Analyses on the Effect of Growth Mindset Manipulation on Growth Mindset

Section A. Participant Exclusion.

Of the 199 participants who consented to take part in the survey, we first excluded 22 because they received a completion message at the beginning of the survey because of experimenter error (N = 199-22 = 178). We then conducted four quality checks, 1) whether the participant reported the same gender they identify with in the screener survey before the study and the demographic questionnaire at the end of the study, 2) whether their child was between the ages of 3 and 5 based on the child's date of birth participants reported, 3) whether the child's age at which parents started reading to them, as reported in the literacy home learning environment questionnaire, was younger than the child's age calculated based on the reported date of birth, and 4) whether participants answered as least three of the six attention check questions correctly. Thirty-five participants did not pass these quality checks and were excluded (N = 178 – 35 = 143). Finally, we coded participants' summaries of the mindset induction or the control articles on whether they were consistent with the main points of the article assigned to that participant. Twenty-three participants did not pass this manipulation check and were excluded (N = 143 – 23 = 120). These 120 participants were our analytic sample.

Section B. Screener Survey

1. Are you the caregiver of a pet that lives with you? If so, what kind? (If you have more than one kind of pet, check all that apply.)
   - o Yes, cat(s)
   - o Yes, dog(s)
   - o Yes, other
   - o No
   - o I prefer not to answer

2. What gender do you identify with?
   - o Male
   - o Female
   - o Other ______________________________
   - o I prefer not to answer

3. How old are you?
   - o Under 20 years old
   - o 20 - 29 years old
   - o 30 - 39 years old
   - o 40 - 49 years old
   - o 50 years old or older
   - o I prefer not to answer

4. Are you the parent/guardian of a child who lives with you? If so, how old is the child? (If you have more than one child, check all that apply.)
   - o Yes, under 3 years old
   - o Yes, between 3 and 5 years old
   - o Yes, between 6 and 10 years old
   - o Yes, over 11 years old
   - o No, I am not a parent/guardian of a child who lives with me
   - o I prefer not to answer

Section C. Pre-Induction Questionnaires
In this survey, we will ask you to think about your 3- to 5-year-old child when answering all the questions. If you have more than one child in this age range, think about the oldest child in this age range during the survey.

When is your child's date of birth? __Year ___Month

What is your child's gender?

- o Boy
- o Girl
- o Other

Home Learning Environment - Book Title Checklist
Blow you will see a list of 60 titles.
Some of these are titles of popular children's books and some are made up.
Please read the titles and check the box in front of those names which you know to be titles of children's books. Do not guess, but only check those you know.

A Difficult Day
Big Old Trucks
How Stephen Found a Pet
This is My Family
How Wishes Come True
Busiest Firefighters Ever
The Snowy Day
Caps for Sale
Zack's House
Hello Morning, Hello Day
Franklin in the Dark
The Shy Little Kitten
Go Dog Go
What Do I Hear Now?
Mortimer
Goodnight Moon
Martha Rabbit's Family
The Whispering Rabbit
Green Eggs and Ham
Clarissa's Patch
Happy Birthday Moon
Curious George
Harry the Dirty Dog
In the Night Kitchen
Worry No Longer
I Was So Mad
Rachel's Real Dilemma
Jelly Belly

The Velveteen Rabbit
Three Cheers for Gloria
Love You Forever
Matthew and the Midnight Tow Truck
Terry Toad
Alexander and the Terrible, Horrible, No Good, Very Bad Day
Murmel, Murmel, Murmel
The Toy Truck
Polar Express
Red is Best
Saggy Baggy Elephant
I Hear a Knock at My Window
Scuffy the Tugboat
The Poky Little Puppy
A Pocket for Corduroy
Kimberly's Horse
Snowflakes Are Falling
The Runaway Bunny
Winter Fun on Snowy Days
Just Me and My Dad
Bears on Wheels
The Very Hungry Caterpillar
The Wonderful Pigs of Jillian Jiggs
Thomas' Snowsuit
Tootle
Alligator Pie
Farmer Joe's Hot Day
Eleanor and the Magic Bag
We're Going on a Bear Hunt
The Paper Boat's Trip
Where the Wild Things Are
Tracy Tickles
I don't recognize any of these titles

Home Learning Environment - Spatial Toys and Activities Checklist
Blow you will see a list of 60 names.
Some of these are names of popular children's games and toys and some are made up.
Please read the names and check the box in front of those names which you know to be names of children's games and toys. Do not guess, but only check those you know.

Blokus
Dominoes
Etch A Sketch
K'Nex
Snapweez

Inkthink
LEGO
Snakey Scramble
Knotbots
Lincoln Logs
Tank Bank
Mega Bloks
Airfliers
Minecraft
Tinker Toy
Craterfrogs
Magna-Tiles
Pattern Blocks
Quipitz
Ravensburger
Magformers
Rubik's
Snap Circuits
Braintacs
Unifix Cubes
Tangram
Zoobtoob
Duplos
Mindware Marble Run
Critmobiles
Botzees
Brickyard
Geomag
Shape Mags
Bloco
Clicformers
Goobi
CitiBlocs
KangaBlox
Maze Balls
Rinx
Tegu
Lite-Brite
Enginvent
SmartMax
Plus-Plus
Shop Straws
Linderhop
Mega Construx

Nanoblock
Mental Blox
Zoob BuilderZ
Engino
Bristle Blocks
Funcubez
Edushape
Fat Brain Toys
Clock-Tocks
Leapers
Brainfeeder
I don’t recognize any of these names

Home Learning Environment - Literacy Questionnaire

The following questions will ask about the activities you may do at home with your child.

1. At bedtime, how often do you, or other members of the family, read to your child?
   - Never
   - Once a month
   - 2-3 times a month
   - 1-2 times a week
   - 5-6 times a week
   - Daily
   - I prefer not to answer
2. At other times, how often do you, or other members of the family, read to your child?
   - Never
   - Once a month
   - 2-3 times a month
   - 1-2 times a week
   - 5-6 times a week
   - Daily
   - I prefer not to answer
3. How often does your child ask to be read to?
   - Never
   - Once a month
   - 2-3 times a month
   - 1-2 times a week
   - 5-6 times a week
   - Daily
   - I prefer not to answer
4. How often does your child go to the library? (Please answer this question based on your experience before the COVID-19 pandemic.)
   - Never
   - Once a month
   - 2-3 times a month
   - 1-2 times a week
   - 5-6 times a week
   - Daily
   - I prefer not to answer
5. How many children's books are available in your household?
   - None
   - 1-20
   - 21-40
   - 41-60
   - 61-80
   - More than 80, please estimate ______________________
   - I prefer not to answer

6. How old was your child when you started reading picture books to him or her?

__ years __months

Home Learning Environment - Spatial Questionnaire
How frequently do you engage in the following activities with your child? (All questions used the same scale. Only shown for the first question.)

1. Play with Puzzles (e.g., picture puzzles, tangrams).
   - Never
   - Once a month
   - 2-3 times a month
   - 1-2 times a week
   - 5-6 times a week
   - Daily
   - I prefer not to answer
2. Do mazes.
3. Do connect the dots activities.
4. Build with construction toys (e.g., building blocks, LEGOs, magnet sets, Lincoln Logs).
5. Play computer games, apps or visit interactive websites that involve building things.
6. Draw maps or floor plans.

Beliefs about Child's Ability, Interest, and Importance of Child's Ability – Literacy
This questionnaire asks some questions about verbal activities, such as reading storybooks or telling stories.

1. How good is your child at verbal activities?
   - (Not Good At All)
   - 2
   - 3
   - 4
   - 5
   - 6
   - 7 (Very Good)
   - I prefer not to answer
2. How much does your child like verbal activities?
   - (Not Very Much)
   - 2
   - 3
   - 4
   - 5

- 6
- 7 (Very Much)
- I prefer not to answer

3. How important is it to you that your child does well at verbal activities?
    - 1 (Not Very Important)
    - 2
    - 3
    - 4
    - 5
    - 6
    - 7 (Very Important)
    - I prefer not to answer

Beliefs about Child's Ability, Interest, and Importance of Child's Ability - Spatial

This questionnaire asks some questions about spatial activities, such as doing puzzles or building with blocks. (Scales were the same as the corresponding questions in the verbal domain.)
How good is your child at spatial activities?
How much does your child like spatial activities?

How important is it to you that your child does well at spatial activities?

Belief about Own Ability – Literacy

This questionnaire consists of several statements about your verbal abilities, preferences, and experiences. After each statement, you should select a number to indicate your level of agreement with the statement. (All questions used the same scale. Only shown for the first question.)

1. I am good at crossword puzzles.
    - 1 (Strongly Disagree)
    - 2
    - 3
    - 4 (Neither Agree/Disagree)
    - 5
    - 6
    - 7 (Strongly Agree)
    - I prefer not to answer
2. I am good at Scrabble.
3. I often have trouble finding the right word to say.
4. I would rather read a text explanation than look at a drawing or figure.
5. I have a good vocabulary.
6. I spend more time reading than most people I know.
7. I prefer to watch TV or movies than to read for leisure.
8. I can easily follow a complex verbal argument.
9. I often have trouble expressing what I mean in words.
10. I have a good sense of language usage and write grammatically.

Belief about Own Ability – Spatial

This questionnaire consists of several statements about your spatial abilities, preferences, and experiences. After each statement, you should select a number to indicate your level of agreement with the statement. (All questions used the same scale. Only shown for the first question.)

1. I am good at determining if my car fits into an available parallel parking spot.
   - 1 (Strongly Disagree)
   - 2
   - 3
   - 4 (Neither Agree/Disagree)
   - 5
   - 6
   - 7 (Strongly Agree)
   - I prefer not to answer
2. I always know if a chair will fit through my front door before buying it.
3. I can easily visualize my room with a different furniture arrangement.
4. I enjoy putting together puzzles.
5. I can easily visualize the location of electrical sockets along the other side of the wall in the adjoining room to my bedroom.
6. I am good at putting together furniture with only the use of diagrams.
7. I have trouble giving someone directions, using a map they are holding, without the ability to rotate the map to match the direction I am currently facing.
8. I can easily fold an elaborate paper airplane using a diagram.
9. I can easily imagine what a 3D landscape would look like from a different point of view.
10. I can clearly imagine how snow would accumulate in a courtyard on a windy day.
11. I can clearly imagine how water flows through a rocky landscape.
12. I can easily recreate an origami piece after watching someone else make it.
13. I can visualize what the cut face of an apple would look like when the apple is cut on different planes.
14. I could clearly imagine what a soda can would look like after it was partially crushed.
15. I have a hard time recognizing a familiar place from a satellite image.
16. I would be very good at building a model airplane, car, or train.

Mindset – Literacy

Please indicate the extent to which you agree or disagree with each of the following items. By verbal ability, we mean your child's ability to learn language-related knowledge and skills, not his or her current language knowledge and skills. (All questions used the same scale. Only shown for the first question.)

1. My child's verbal ability is innate and will never change.
   - 1 (Strongly Disagree)
   - 2
   - 3
   - 4 (Neither Agree/Disagree)
   - 5
   - 6
   - 7 (Strongly Agree)
   - I prefer not to answer

2. My child's verbal ability can change significantly from birth.
3. After a certain point in childhood, my child's verbal ability cannot improve.
4. My child can always improve his/her verbal ability, no matter how old he/she is.
5. My child's verbal ability can only be substantially improved during a specific period of time in his/her development.
6. My child is past the age at which he/she can substantially improve his/her verbal ability.

Mindset - Spatial

Please indicate the extent to which you agree or disagree with each of the following items. By spatial ability, we mean your child's ability to learn spatial knowledge and skills (e.g., to understand the spatial relations among objects, to build blocks, and to solve puzzles), not his or her current spatial knowledge and skills. (All questions used the same scale. Only shown for the first question.)

1. My child's spatial ability is innate and will never change.
   - 1 (Strongly Disagree)
   - 2
   - 3
   - 4 (Neither Agree/Disagree)
   - 5
   - 6
   - 7 (Strongly Agree)
   - I prefer not to answer
2. My child's spatial ability can change significantly from birth.
3. After a certain point in childhood, my child's spatial ability cannot improve.
4. My child can always improve his/her spatial ability, no matter how old he/she is.
5. My child's spatial ability can only be substantially improved during a specific period of time in his/her development.
6. My child is past the age at which he/she can substantially improve his/her spatial ability.

Section D. Manipulation Articles
Now we'd like you to read a report on some recent research articles. You'll want to understand the main point of it as there will be a short memory and comprehension test afterward.

Growth Mindset Induction

The Origins of Intelligence:
Is the Nature-Nurture Controversy Resolved?
Posted Mar 06, 2018, by Jerome M. Berglund, Ph.D.

Adam Steagal is gifted. Although he is just eighteen months old, he can understand over 2000 words, has a speaking vocabulary of 500 words, and is even able to identify five different species of birds. Early in his life, Adam's parents had a hunch that he was unusual.

At the age of 8 months he was crawling and investigating everything in the Steagal household. All babies are curious, but Adam's curiosity led him to heights of baby creativity. He was not simply banging on pots and pans; Adam had learned to dismantle a toy camera and put it back together again. He had the coordination to handle small objects, the ability to remember how parts fit together, and could concentrate on the camera for almost an hour. Most children can't do what Adam was doing until they are at least three or four. When he was ten months old, Adam's parents brought him to Yale University's Unit for Intelligence Research (UIR). Paula Rescorla, the director of UIR, found that Adam had an IQ of 185. Experts consider an IQ of 130 "very superior". Adam's IQ is so extreme that only one person in a million has an IQ that even comes close. Researchers like Rescorla are keenly interested in what makes Adam so smart.

The traditional "is it heredity or is it environment?" question is battered around the halls of UIR daily. Yet, people who take the side that intelligence is genetically determined are going to be believed less and less; current research shows that intelligence can be increased substantially by environmental factors.

"The brilliance of Leonardo da Vinci and Albert Einstein was probably due to a challenging environment. Their genius had little to do with their genetic structure."

In the past decade, a number of comprehensive studies have been published in the United States and in Europe. These studies provide the clearest answers so far in the ongoing debate. The most significant of these studies will be published this fall in Psychological Review, a prestigious psychological journal published in the United States. John Knowles, the author of the article and a professor at Harvard, concludes that, "Intelligence seems to have a minimal genetic component. People may be born with a given level of intelligence, but we see increases in IQs up to 50 points when people enter stimulating environments."

Knowles spent the last decade tracing identical twins who were raised apart. In a relentless search through Latin America, Africa, and North America, he was able to locate 83 pairs of twins who were raised separately. These twins ranged in age from 7 to 51 and came from all

economic levels. Knowles had an ideal sample to study the nature-nurture question. The twins in his study were often reared in different places by parents with different circumstances. The various pairs of twins came from different countries, spoke different languages, were different ages, and he followed them for ten years. Knowles tested the subjects individually with the best “culture-fair” intelligence tests available.

Culture-fair tests measure intelligence by having people identify relationships between shapes and objects. Because the tests use only shapes and objects - not words - to measure intelligence, cultural factors, like language, don’t influence people’s scores. Consequently, they provide a much more accurate measure of intelligence than most other intelligence tests. In addition, culture-fair tests don’t discriminate against any ethnic groups. Because Knowles used these sophisticated measures of intelligence, he was able to make stronger conclusions than have been possible in the past.

He found that twins raised in different environments had very different levels of intelligence. According to his results, up to eighty-eight percent of a person’s intelligence is due to environmental factors. In his study, twins raised in stimulating environments with motivated parents tended to have high IQs, whereas twins raised in unstimulating environments tended to have lower IQs. As an example, a child raised in a family that spends lots of time reading books, doing puzzles, and talking together may have very different outcomes than a child who does not experience these activities.”

Although this evidence is very strong, Knowles has even more evidence which may convince skeptics. He found that people in challenging environments showed substantial increases in their intelligence during the ten year study. Children and adults who were in stimulating environments had increases in IQ ranging from 15 to 48 points. People who were in unstimulating environments showed slight drops in their IQ. According to Knowles, his results suggest that “the brilliance of Leonardo da Vinci and Albert Einstein was probably due to a challenging environment. Their genius had little to do with their genetic structure. These men are truly admirable because they were challenged and worked to overcome obstacles.”

Other researchers are finding similar results. Howard Gardner recently published an article supporting Knowles’ research. Gardner’s studies show that person’s level of motivation can have a profound effect on intelligence. He found that bright children placed in “dull” environments tended to become less intelligent unless they were motivated to learn. Relatively dull children placed in stimulating environments seemed to get much smarter, especially if they were rewarded for learning new things. Gardner said, “I spent much of my life believing intelligence was genetically determined. Now my research suggests that people do not have superior genes.”

"The best available research shows that intelligence can be increased by stimulating environments."

Needless to say, Knowles’ and Gardner’s research is drawing much attention from other psychologists. Their findings are widely praised by researchers who have been trying for years to prove that intelligence is not genetically determined. Leo Kamin of Princeton University is one such researcher. In the 1960s and ‘70s, he argued strongly that there was no good evidence to

show the link between intelligence and genetics. He helped prove that Sir Cyril Burt, a now infamous researcher, faked his data to show that intelligence was inherited. When Burt was alive, the Queen of England knighted him for his "brilliant" research. When Kamin examined Burt's results, he discovered serious flaws that could only have resulted by faking the data.

This has led Kamin to be a bit careful before accepting any intelligence findings as the "truth". Consequently, he carefully examined Knowles' study. He says he found "no flaws in [Knowles'] methods or his analysis. Finally, the best available research shows what I have been arguing for for 25 years. Knowles' research is simply the best, and it shows that intelligence can be increased by stimulating environments."

Paula Rescorla at Yale's UIR is also excited about Gardner's and Knowles' results. "I think the absolutely critical thing that has come out of these studies is that intelligence is something that motivated people can acquire. I think this idea will revolutionize education in the coming years. We can help motivated children find environments that will help them increase their abilities."

The eighteen month-old genius Adam Steagal seems to be in an ideal environment right now. His young brilliance is being challenged by fascinating toys and games. But apparently, whether he will be brilliant when he grows up is largely up to his environment.

Jerome M. Berglund, Ph.D. is a professor at the University of Colorado. He is a frequent contributor to Psychology Today.

Control - Déjà Vu

Déjà vu Linked to Feelings of Prediction

New research demonstrates an illusory sense of prediction during déjà vu.

Posted Mar 06, 2018, by Anne M. Cleary, Ph.D.

Déjà vu—the strange feeling of having been to this very place or done this very thing before when you know you haven't—happens to most people at some point in their lives. But did you know that for many people, this feeling is accompanied by a feeling of knowing what will happen next? Maybe this even happens to you sometimes. New research is finally shedding light on this strange association.

For many years, I have approached déjà vu from the perspective that it is a memory phenomenon—that déjà vu occurs because something relevant to the current situation resides in our memory but we fail to call it to mind. The concept is humorously illustrated in this commercial from years back, where a man experiences deja vu upon entering his hotel room for the first time, and his partner points out that it is only because he had previously done the virtual tour at hotels.com.

However, for many people, the experience of déjà vu is more than just a feeling of a memory. For many people, déjà vu is also a feeling of knowing what will happen next. For them, the

experience illustrated by the hotels.com commercial seems incomplete. Their experience might instead additionally include a feeling of knowing what was around the corner in the room.

“However, for many people, the experience of déjà vu is more than just a feeling of a memory. For many people, déjà vu is also a feeling of knowing what will happen next.”

In my 2017 TEDxCSU talk, I described a phone call from a man in Alaska who was searching for information that might explain this type of freaky mind-boggling experience of premonition during deja vu. His experience really bothered him because he wasn't superstitious and couldn’t explain it, and he’d been searching the web for explanations. At numerous presentations on déjà vu over the years, my students and I have heard similar stories from people about their experiences with déjà vu.

“Is there something to this?” I wondered.

Digging into old research, hints of an association between déjà vu and feelings of prediction could indeed be found. In a 1959 publication, Mullan and Penfield describe electrically stimulating a patient’s brain in a way that not only induced déjà vu in the patient, but also a feeling of knowing exactly what was going to happen next. The patient reported knowing exactly what the doctor would say and do next during the electrically-induced déjà vu experience.

In this case, the feeling of prediction during deja vu was illusory. But might it be possible for the feeling to be accurate if it is rooted in a memory?

Thinking back to the man in the hotels.com commercial, if the man's initially mind-boggling sensation of déjà vu was rooted in memory, might his unretrieved memory for his virtual tour could also drive a mysterious sense of prediction regarding what was around the bend in the room?

Anecdotally, some people even have examples where they do seemed to have been able to predict, and it turns out to have likely been rooted in an initially unretrieved memory. In September, 2016, a famous memory researcher—Dr. Elizabeth Loftus—was visiting Colorado State University to give a talk on our campus. In conversing briefly about some of my work on déjà vu, she told an interesting story about something that had happened to her. She was visiting someone’s house for the first time (or so she thought). She walked in and had a strange sense of déjà vu. She also felt that she knew what was behind an interior window within in the home—like she’d been there before and knew. Later on, she discovered she had been there. Years earlier, she had attended a wedding at that very house as a guest of a friend, but failed to recall that when first walking in. In a strange coincidence, she was now meeting the homeowner, and in an entirely different context.

Many notable memory researchers have suggested that the real adaptive purpose of memory is to predict the future from our past experiences. Might this explain the strange association between deja vu and feelings of prediction?

“Many notable memory researchers have suggested that the real adaptive purpose of memory

is to predict the future from our past experiences."

We examined this in our new study recently published in Psychological Science. In it, we built on previous work that showed that spatial similarity of a new scene to an unrecalled scene in memory can contribute to déjà vu reports. We had participants view videos that were much like taking virtual tours through rooms and landscapes. For each scene, participants were taken through a series of turns while on the tour. Later, they virtually toured new scenes, some of which spatially mapped onto an earlier-toured scene and followed the same navigational path as in that earlier scene—up to a point. At that point, the movement stopped short of the next turn. We wanted to see if participants would feel a sense of knowing the direction of the next turn when experiencing déjà vu, and if any such sense would be accompanied by actual predictive ability (that was rooted in memory for the direction of the turn taken in the earlier-viewed but unrecalled spatially similar scene).

We found that there was an association between reports of déjà vu and feelings of prediction. During déjà vu reports, people reported stronger feelings of being able to predict the direction of the next turn than when they were not experiencing déjà vu. However, the feeling of prediction turned out to be illusory. Participants' ability to select the correct turn during déjà vu was at the level of random guessing.

Why should déjà vu be accompanied by an illusory sense of prediction? We surmise that, in much the same way that a tip-of-the-tongue state feels as if retrieval of a word is imminent, the déjà vu state may feel as if retrieval of the current situation is imminent--as if it isn't new and the whole thing is about to come to mind. If so, it makes sense that a person might also feel that retrieving how this whole event unfolds is also imminent—maybe it feels like whatever is around the next bend is just about to come to mind. This could conceivably give rise to a feeling of prediction. But, there is a difference between feeling as if one is about to access something from memory and actually accessing it in time to predict what is going to happen.

So, if you've ever experienced a feeling of prediction during déjà vu, you're not alone. It appears from our work to be a common experience. However, be skeptical of your actual ability to predict when you have this feeling—your mind may be tricking you.

Anne M. Cleary, Ph.D. is a professor at the University of Colorado. She is a frequent contributor to Psychology Today.

Manipulation Check

1. In the space below, please briefly describe the main points of the article you read.

   ____
   ____
   ____
   ____
   ____

2. How difficult to understand did you find the article to be? (The following four questions used the same scale. Only shown for the first question.)

- 1 (Not At All)
- 2
- 3
- 4
- 5
- 6
- 7 (Very Much)
- I prefer not to answer

3. How credible did you find the article to be?
4. How persuasive did you find the article to be?
5. Please rate the degree to which you agree with the views expressed in the article.

Section E. Post-Induction Questionnaires

Learning Activity Choices

For the following questions, please select one game or activity out of the three options that you would choose to play with your child today. It does not need to be a game or activity you have at home, just choose which activity you would play with your child.

1. Which book would you read with your child?

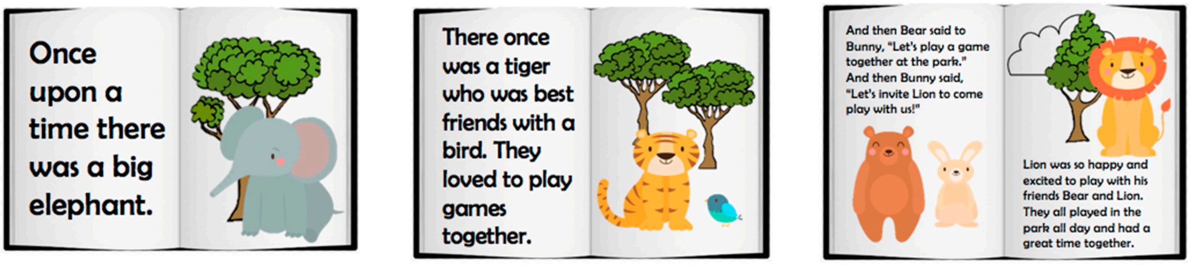

A B C

2. With LEGOs like these, which structure would you try to build with your child?

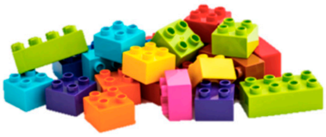

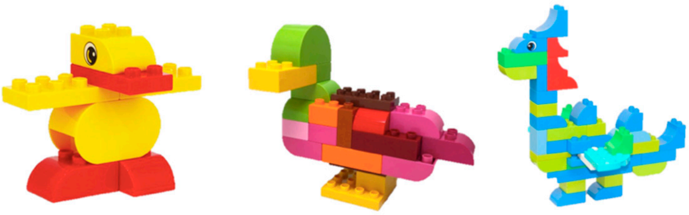

A B C

3. Which topic would you use to start a conversation with your child at dinner?

A	B	C
"Do you like your dinner?"	"What was your favorite part of your day today?"	"What are you most excited to do tomorrow?"

4. Which book would you check out at the library with your child?

10 Pages 20 Pages 30 Pages

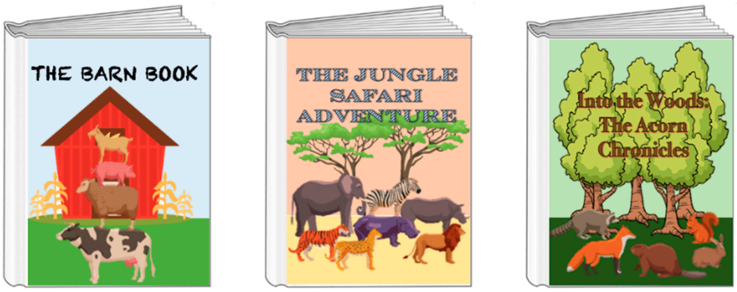

A B C

5. With magnet tiles like these, which structure would you try to build with your child?

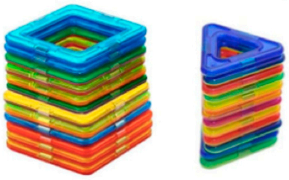

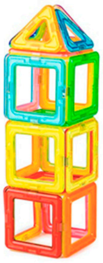

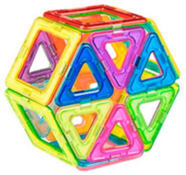

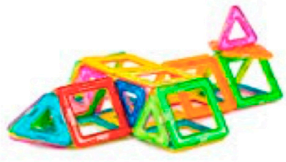

A B C

6. Which words would you practice reading with your child?

A	B	C
the is we	cow big play	moon apple bird

7. With blocks like these, which structure would you try to build with your child?

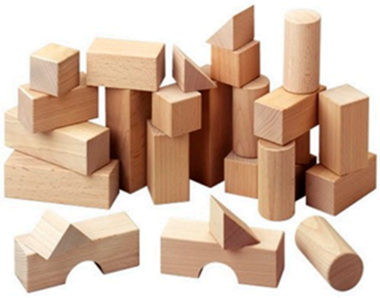

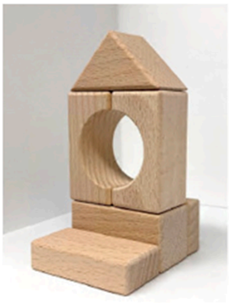

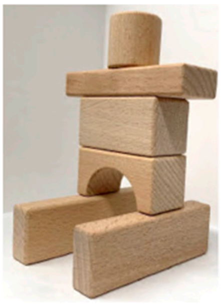

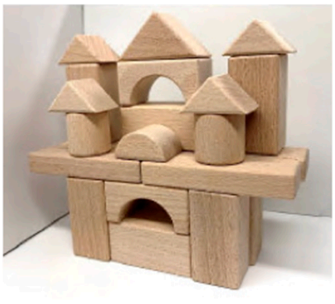

A B C

8. Which of these pictures would you try and use to create a story with your child?

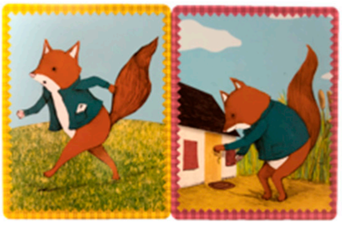

A

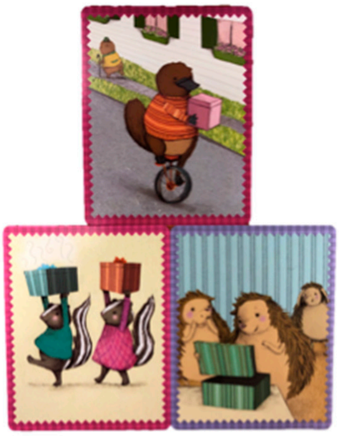

B

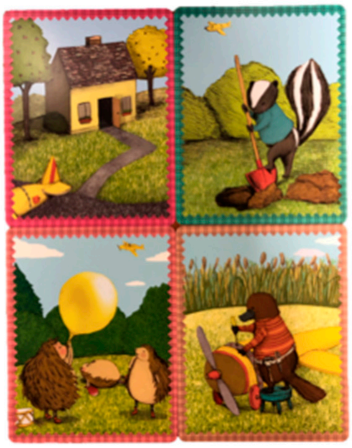

C

9. Which puzzle would you try to put together with your child?

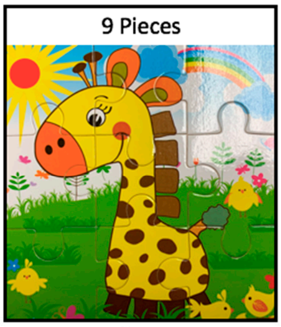

A

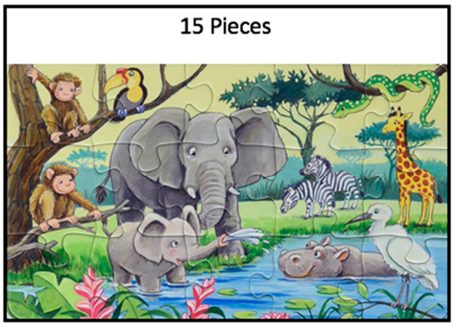

B

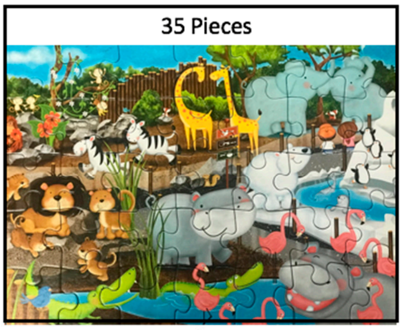

C

10. With pattern blocks like these, which pattern would you try to make with your child?

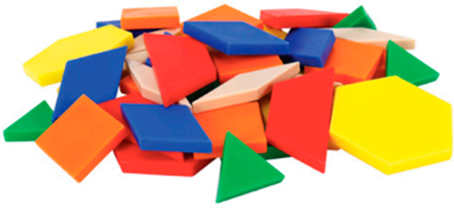

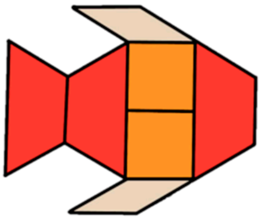

A

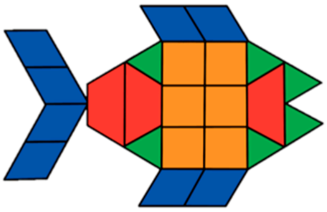

B

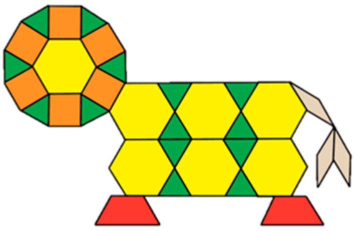

C

11. Which letters or words would you practice sounding out with your child?

A	B	C
a b c d e f<br>g h i j k	mat hat<br>cat	tree bike<br>down

12. Which maze would you choose to do with your child?

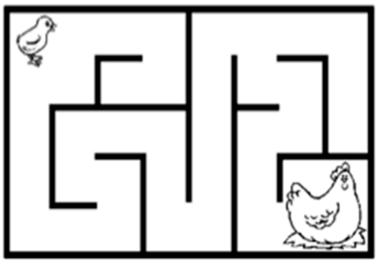

A

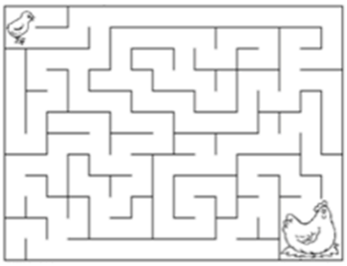

B

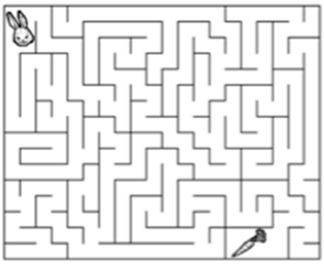

C

Activity Choice Considerations
When choosing toys and games to play with your child, rate the importance of the following aspects on a 1 – 7 scale, with 1 being not important at all, and 7 being extremely important.

1. My child's enjoyment
2. My own enjoyment
3. How hard it is for my child
4. How hard it is for me
5. How much my child can learn from it

Are there any other aspects you would consider when choosing toys and games to play with your child?

When you play toys and games with your child, which level of difficulty would you choose?
Very Easy In the Middle Very Hard
0 50 100

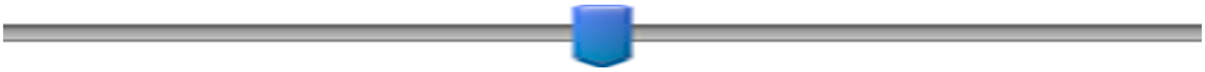

On a 1 – 7 scale, rate how important the following activities are for improving your child's verbal ability, with 1 being not important at all, and 7 being extremely important.

1. Playing verbal games at home
2. Parents' teaching about verbal skills
3. Learning verbal skills from school

On a 1 – 7 scale, rate how important the following activities are for improving your child's verbal ability, with 1 being not important at all, and 7 being extremely important.

1. Playing spatial games at home
2. Parents' teaching about spatial skills
3. Learning spatial skills from school

Mindset – Literacy

Same as in pre-induction measures.

Mindset – Spatial

Same as in pre-induction measures.

Section F. Criteria to determine difficulty for each activity on the Learning Activity Choice questionnaire.

Item	Difficulty Criterion	Choice A	Choice B	Choice C
Which book would you read with your child?	Item Image	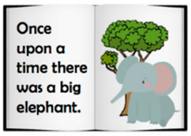	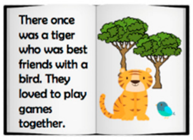	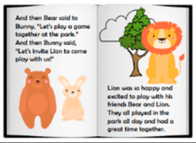
	number of words	9	18	54
With LEGOs like these, which structure would you try to build with your child?	Item image	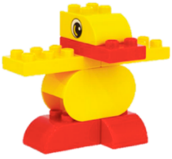	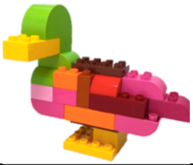	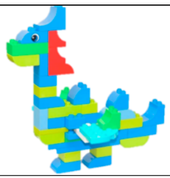
	number of blocks	7	19	37
Which topic would you use to start a conversation with your child at dinner?	Item image	"Do you like your dinner?"	"What was your favorite part of your day today?"	"What are you most excited to do tomorrow?"
	number of words	5	9	8
	time	now	today	future
Which book would you check out at the library with your child?	Item image	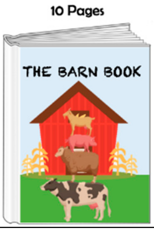	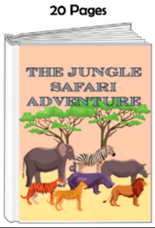	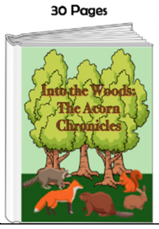
	number of pages	10	20	30
With magnet tiles like these, which structure would you try to build with your child?	Item image	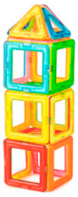	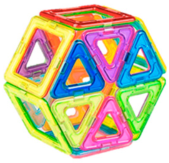	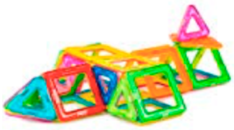
	number of blocks	19	18	27
	Visual complexity	Low	Medium	High

Which words would you practice reading with your child?	Item image	the is we	cow big play	moon apple bird
	Average number of letters in each word	2.33	3.33	4.33
With blocks like these, which structure would you try to build with your child?	Item Image	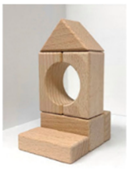	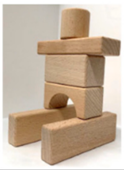	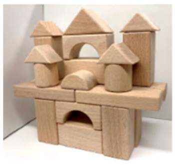
	number of blocks	6	6	21
	Balancing requirement	Low	Medium	High
Which of these pictures would you try and use to create a story with your child?	Item Image	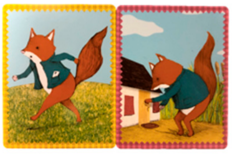	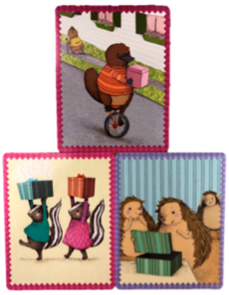	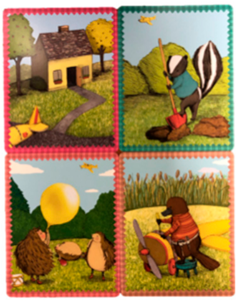
	number of cards	2	3	4
Which puzzle would you try to put together with your child?	Item Image	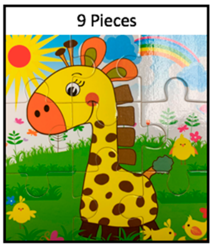	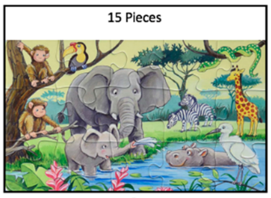	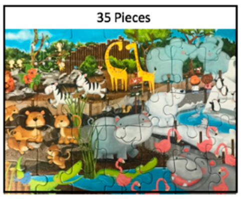
	number of pieces	9	15	35
With pattern blocks like these, which pattern would you try to make with your child?	Item Image	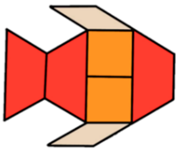	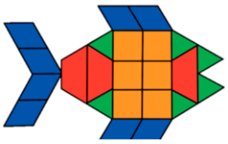	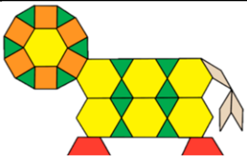
	number of blocks	7	23	32

Which letters or words would you practice sounding out with your child?	Item image	a b c d e f g h i j k	mat hat cat	tree bike down
	number of letters in each word	1	3	4
Which maze would you choose to do with your child?	Item image	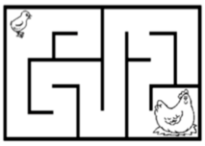	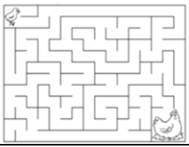	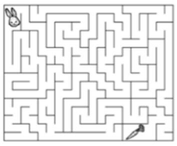
	number of grids	8 x 5	16 x 12	20 x 16

Section G. Histograms of participants' responses on the HLE and the learning activity choice measures, by condition

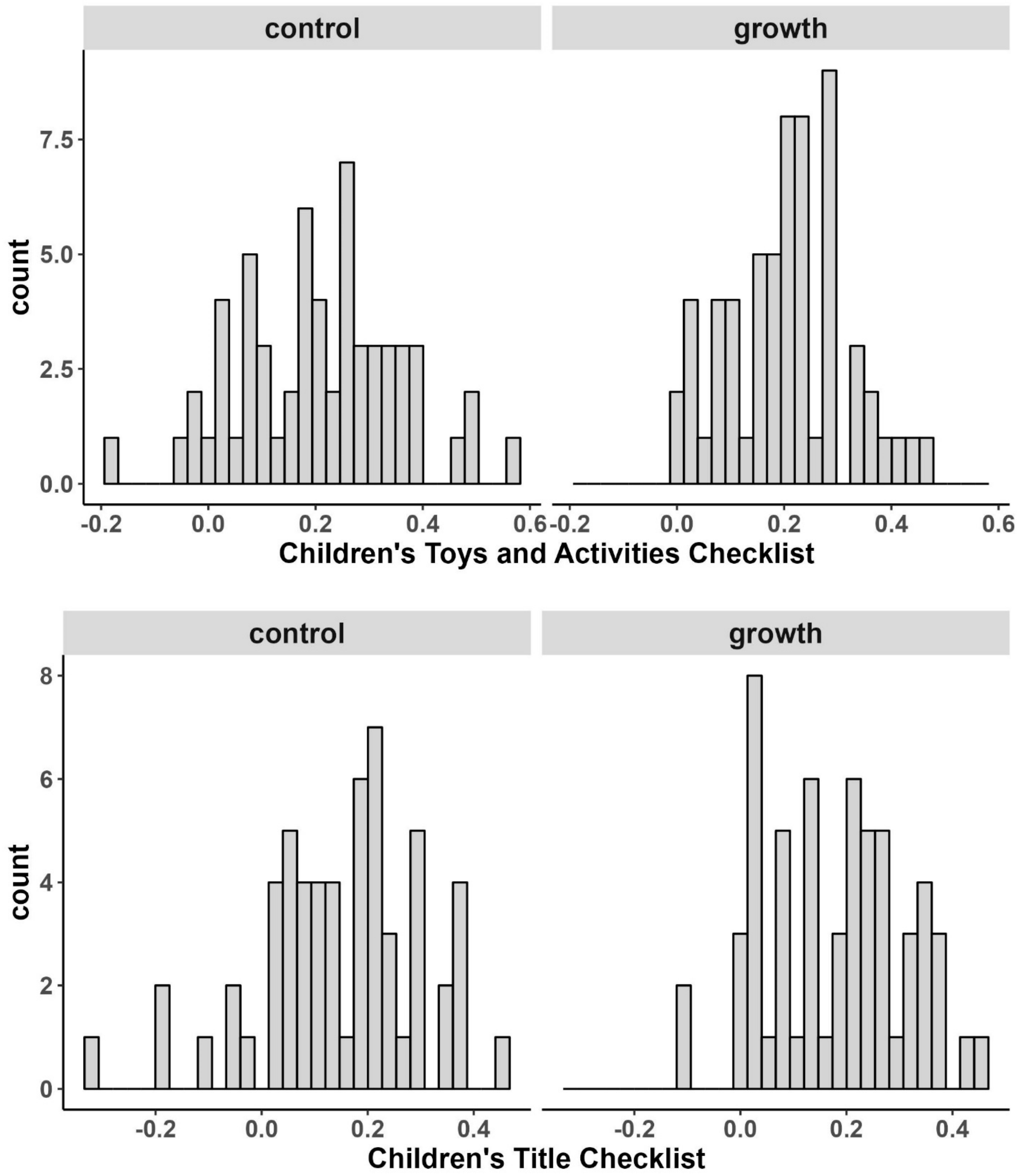

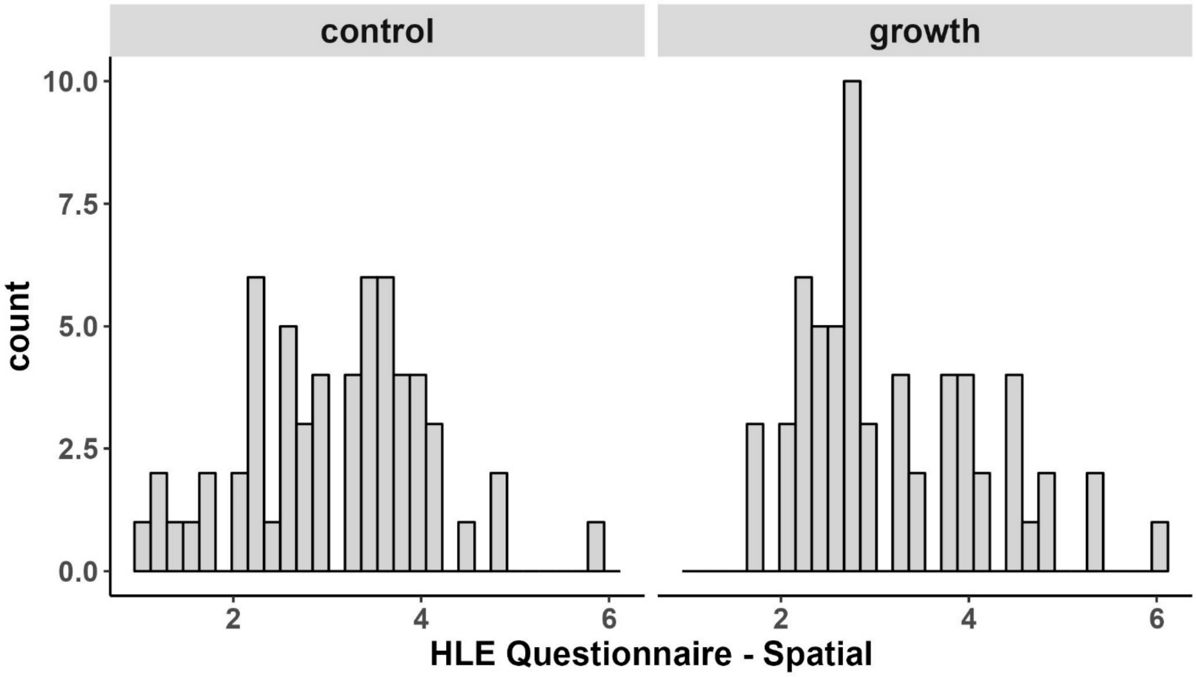

control
growth
10.0
7.5
5.0
2.5
0.0
count
2
4
6
HLE Questionnaire - Spatial

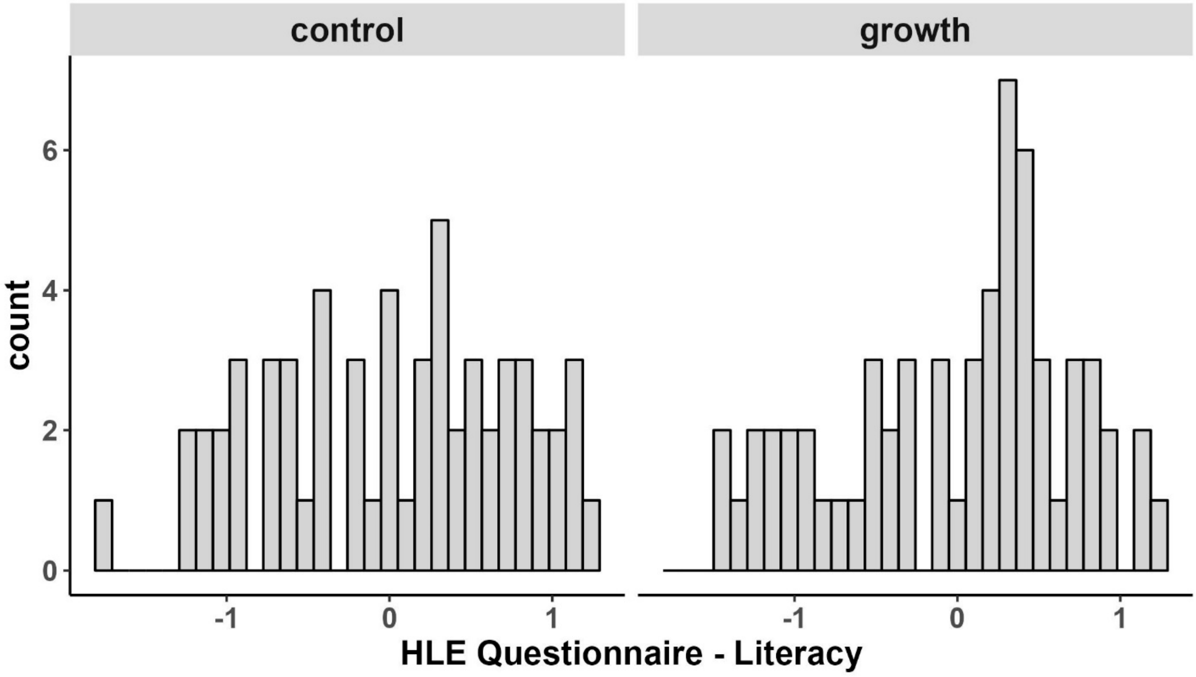

control
growth
6
4
2
0
count
-1
0
1
HLE Questionnaire - Literacy

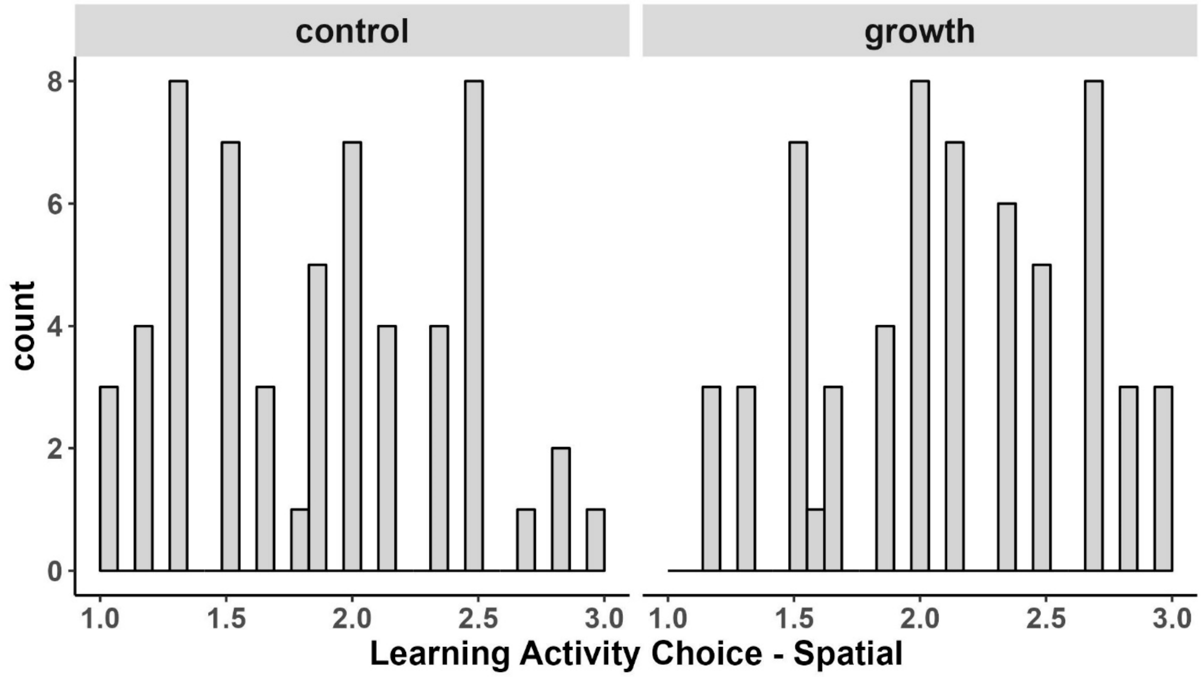
control
growth
count
0
2
4
6
8
1.0
1.5
2.0
2.5
3.0
Learning Activity Choice - Spatial

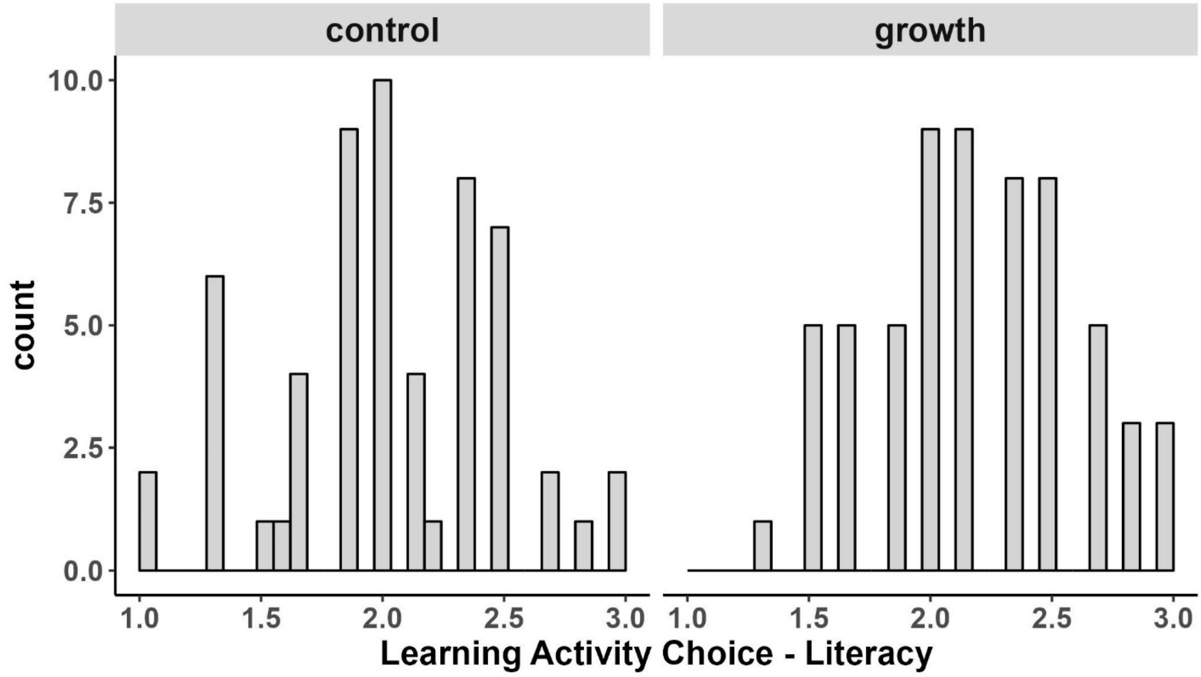
control
growth
count
0.0
2.5
5.0
7.5
10.0
1.0
1.5
2.0
2.5
3.0
Learning Activity Choice - Literacy

Section H. Correlations Among Items on Spatial Home Learning Environment Questionnaire and Spatial Toys and Activities Checklist

	1	2	3	4	5	6
1. Puzzle						
2. Maze	0.404***					
3. Connect Dot	0.324***	0.617***				
4. Construction	0.337***	0.267**	0.222*			
5. Maps/Plans	0.194*	0.404***	0.501***	0.022		
6. HLE QRE - S	0.665***	0.796***	0.783***	0.553***	0.605***	
7. STAC	0.229*	-0.08	-0.185*	0.245**	-0.361***	-0.041

Note. HLE QRE – S stands for spatial home learning environment questionnaire. STAC stands for Spatial Toys and Activities Checklist. * $p < .05$, ** $p < .01$, *** $p < .001$.

Section I. Results of regression models predicting learning activity choices separately for the spatial and literacy domains.

We ran a series of regression model to examine whether reading the growth-mindset article led participants to choose more challenging activities, separately for the spatial and literacy domains. Similar to the models reported in the main text, we first entered condition (growth-mindset vs. control), and demographic factors (child gender, SES, and child age) and gradually added other pre-test measures. Tables 1S and 2S shows results of these models.

Table 1S. Results of Linear Regressions Predicting Challenge Level of Activity Choices (Std. Beta Coefficients) in the Spatial Domain.

	Model 1S	Model 2S	Model 3S	Model 4S
Intercept	-0.39 *	-0.42 *	-0.40 *	-0.41 *
Condition: growth	0.47 *	0.46 *	0.47 *	0.47 *
Child gender: boy	0.26	0.31-	0.27	0.30
SES	0.11	0.09	0.09	0.07
Child age	0.08	0.08	0.07	0.08
HLE Questionnaire		0.11	0.13	0.08
Checklist		0.23 *	0.17	0.21
Pre-Induction Growth Mindset			0.11	0.08
Belief about Child Ability			0.09	0.08
Belief about Child Interest			-0.07	-0.09
Belief about Child Importance			-0.03	-0.06
Belief about Own Ability				0.18
R-squared	0.09	0.15	0.16	0.18
Adjusted R-squared	0.05	0.10	0.08	0.09

Table 2S. Results of Linear Regressions Predicting Challenge Level of Activity Choices (Std. Beta Coefficients) in the Literacy Domain.

	Model 1L	Model 2L	Model 3L	Model 4L
Intercept	-0.05	-0.17	-0.17	-0.01
Condition: growth	0.36 *	0.40 *	0.41 *	0.33 *
Child gender: boy	-0.24	-0.04	-0.04	-0.24
SES	0.13	0.11	0.08	0.10
Child age	0.08	0.10	0.07	0.12
HLE Questionnaire		0.25 *	0.17	0.05
Checklist		0.04	0.02	0.04
Pre-Induction Growth Mindset			0.05	0.03
Belief about Child Ability			0.16	0.11
Belief about Child Interest			-0.01	-0.13

Belief about Child Importance			0.23 *	0.19 *
Belief about Own Ability				0.42 ***
R-squared	0.07	0.15	0.24	0.36
Adjusted R-squared	0.03	0.10	0.16	0.29

Section J. Analyses on the Effect of Growth Mindset Manipulation on Growth Mindset

We examined whether the growth mindset article led to stronger growth mindset, and whether this effect differed by child gender and domain (i.e., spatial vs. literacy). We fit mixed-effect regression models with post-induction growth mindset as the dependent variable. Pre-induction growth mindset, condition, child gender, domain, child age, and family SES were entered as fixed effects and participants were entered as a random effect. The model with interactions among condition, child gender, and domain was not significantly different from the model without these interactions, $\chi^2(4) = 6.06,\ p = .194$. For ease of interpretation, we present results of the model without interaction terms. In this model, only pre-induction growth mindset was a significant predictor, $\beta = 0.79, p < .001$. The effect of condition was not significant, $\beta = 0.14, p = .077$, and therefore, there was no evidence suggesting participants in the growth-mindset condition held a stronger growth-mindset than participants in the control condition after the induction.
